# Mast cell–derived IL-13 as a driver of Crohn disease–associated intestinal fibrosis

**DOI:** 10.1093/ibd/izag127

**Published:** 2026-07-08

**Authors:** Kwestan Safari, Wei Jen Ma, Prabhreet Sekhon, Priya Rai, Susan C Menzies, Tomoaki Hoshino, Anne-Sophie Fratzscher, Andrew Roth, Karen Madsen, Laura M Sly

**Affiliations:** Department of Pediatrics, Division of Gastroenterology, the University of British Columbia and BC Children’s Hospital Research Institute, Vancouver, British Columbia, V5Z 4H4, Canada; Department of Pediatrics, Division of Gastroenterology, the University of British Columbia and BC Children’s Hospital Research Institute, Vancouver, British Columbia, V5Z 4H4, Canada; Department of Pediatrics, Division of Gastroenterology, the University of British Columbia and BC Children’s Hospital Research Institute, Vancouver, British Columbia, V5Z 4H4, Canada; Department of Pediatrics, Division of Gastroenterology, the University of British Columbia and BC Children’s Hospital Research Institute, Vancouver, British Columbia, V5Z 4H4, Canada; Department of Pediatrics, Division of Gastroenterology, the University of British Columbia and BC Children’s Hospital Research Institute, Vancouver, British Columbia, V5Z 4H4, Canada; Department of Internal Medicine, Division of Respirology, Neurology and Rheumatology, Kurume University School of Medicine, Kurume, Fukuoka, 830-0011, Japan; Department of Molecular Oncology, the University of British Columbia and British Columbia Cancer Research Centre, Vancouver, British Columbia, V5Z 1L3, Canada; Department of Molecular Oncology, the University of British Columbia and British Columbia Cancer Research Centre, Vancouver, British Columbia, V5Z 1L3, Canada; Department of Medicine, and Centre of Excellence for Gastrointestinal Inflammation and Immunity Research (CEGIIR), University of Alberta, Edmonton, Alberta, T6G 2H5, Canada; Department of Pediatrics, Division of Gastroenterology, the University of British Columbia and BC Children’s Hospital Research Institute, Vancouver, British Columbia, V5Z 4H4, Canada

**Keywords:** Crohn disease fibrosis, SH2 domain-containing inositol polyphosphate 5'-phosphatase, interleukin-13, mast cells

## Abstract

**Background:**

Crohn disease is a chronic, immune-mediated disease that can lead to intestinal fibrosis, strictures, and bowel obstruction. The Src homology 2 domain-containing inositol polyphosphate 5′-phosphatase-deficient (SHIP−/−) mouse develops spontaneous Crohn disease–like ileal inflammation and fibrosis. Fibrosis depends on increased phosphatidylinositol 3-kinase p110δ activity downstream of interleukin 4 (IL-4) or IL-13. Because current anti-inflammatory treatments do not prevent or reverse fibrosis, we aimed to identify the key cytokine(s) and its cellular source(s).

**Methods:**

We blocked IL-4 or IL-13 in mice by use of neutralizing antibodies or genetic ablation. Intestinal inflammation and fibrosis were assessed by gross pathology, histopathology, collagen accumulation, muscle thickening, immune cell infiltration, and tissue IL-1β concentrations. *IL13* and its cellular source in stricturing Crohn disease were examined using single-cell RNA sequencing and RNAscope. Mast cells and *Il13* were also assessed in ileal cross-sections from SHIP+/+ and SHIP−/− mice.

**Results:**

Blocking or genetic ablation of IL-13, but not IL-4, significantly reduced ileal fibrosis in mice, including collagen accumulation, smooth muscle thickening, vimentin^ + ^cells, and proliferating vimentin^ + ^cells. Blockade of IL-13 also reduced intestinal inflammation, including immune cell infiltration and ileal IL-1β concentrations. Single-cell RNA-sequencing analyses identified mast cells as the exclusive source of *IL13* in stricturing Crohn disease. Human resection tissues confirmed increased mast cells in diseased small intestine and identified mast cells as the source of *IL13*. SHIP−/− mice similarly showed increased ileal mast cells that were also a source of *IL13*.

**Conclusions:**

IL-13 is critical for intestinal fibrosis in SHIP-deficient mice, and mast cells are a source of *IL13* in Crohn disease–associated intestinal fibrosis.

## Teaser

Short Summary

Interleukin 13 (IL-13) drives intestinal fibrosis in Src homology 2 domain-containing inositol polyphosphate 5′-phosphatase-deficient (SHIP)-deficient mice. Mast cells are sole source of *IL13*, and both mast cells and *IL13* are upregulated specifically in strictured ileal tissues from patients with Crohn disease (CD).

Key Messages: *What is already known?*

Intestinal fibrosis is a common and serious complication of CD, but the cells and signaling pathways driving fibrosis remain unknown.

*What is new here?*

IL-13 acts upstream of PI3Kp110δ to drive intestinal fibrosis in SHIP-deficient mice and blocking or ablating IL-13 reduces fibrosis. Mast cell numbers and *IL13* mRNA are upregulated specifically in strictured tissues from patients with CD, and mast cells are the source of *IL13* in strictured intestinal tissues.

*How can this study help patient care?*

Targeting mast cells or IL-13 may prevent or reduce intestinal fibrosis in people with CD by identifying novel, first-in-kind, therapeutic targets.

## Introduction

Inflammatory bowel disease (IBD) comprises chronic immune-mediated disorders of the gastrointestinal tract, of which Crohn disease (CD) is characterized by transmural inflammation that most commonly affects the terminal ileum.^1^ Intestinal fibrosis is a major complication of CD that can lead to stricture formation, bowel obstruction, and surgery.^1^ Although biological therapies may reduce the incidence of fibrostenotic disease, fibrosis remains a significant clinical problem, particularly in patients in whom medical therapy has failed or who experience postoperative recurrence.^2^^,^^3^ No specific antifibrotic therapy has been approved for intestinal fibrosis, highlighting the need to better define the mechanisms that drive this process.

Mice deficient in the Src homology 2 domain-containing inositol polyphosphate 5′-phosphatase (SHIP) develop spontaneous CD-like inflammation and fibrosis restricted to the distal ileum.^4^ SHIP is a hematopoietic-specific negative regulator of Class I phosphoinositide 3-kinase (PI3K) signaling.^5^ Our laboratory has shown that ileal inflammation in SHIP-deficient mice is driven by increased macrophage PI3Kp110α activity and elevated interleukin 1 beta (IL-1β) concentrations,^6^ whereas fibrosis depends on increased PI3Kp110δ activity and downstream induction of arginase 1 (ArgI) activity.^7^^,^^8^ As such, targeting PI3Kp110δ prevents the development of intestinal fibrosis in this model.^8^

Type 2 cytokines are important regulators of tissue repair and extracellular matrix remodeling.^9^ IL-4 and IL-13 are of particular interest because both signal through receptor complexes containing IL4Rα and can activate PI3Kp110δ downstream of receptor engagement.^5^^,^^9^ IL-4 signals through either the type I receptor (IL4Rα/γc) or type II receptor (IL4Rα/IL13Rα1), whereas IL-13 signals through the type II receptor.^9^ Both cytokines have also been reported to promote collagen synthesis by fibroblasts,^10^^,^^11^ suggesting that they may contribute to intestinal fibrosis.

Thus, we hypothesized that IL-4 and/or IL-13 are targetable drivers of intestinal fibrosis in SHIP−/− mice. Using blocking antibodies and genetic ablation, we showed that IL-13, but not IL-4, is required for the development of intestinal fibrosis and contributes to CD-like ileal inflammation in SHIP-deficient mice. We have further shown that *IL13* is markedly upregulated in stricturing CD, mast cell numbers are increased in stricturing CD and in the ileum of SHIP−/− mice, and mast cells represent a source of *IL13* in stricturing disease in people and of *Il13* in SHIP−/− mice. Finally, we identifyed an *IL13*-expressing mast cell subcluster that is enriched in people with stricturing CD.

## Materials and methods

### Mice

Mice heterozygous for SHIP expression (*Inpp5d+/−*) on a C57BL/6 × 129Sv background (N2) were bred to generate SHIP+/+ (controls) and SHIP−/− mice for experiments. SHIP−/− mice were crossed with mice deficient in IL-4 (IL-4−/−) or IL-13 (IL-13−/−) to generate double deficient mice. The IL-4−/− mice were purchased from the Jackson Laboratory (C57BL/6-Il4^tm1Nnt^/J) and IL-13−/− mice (C57BL/6 background) were a gift from Dr. Tomoaki Hoshino (Kurume University School of Medicine, Japan).^12^ SHIP−/− mice were crossed with IL-4−/− mice to produce SHIP+/+IL-4+/+, SHIP+/+IL-4−/−, SHIP−/−IL-4+/+, and SHIP−/−IL-4−/−. Mice were assessed at 8 weeks of age. SHIP−/− mice were crossed with IL-13−/− mice to produce SHIP+/+IL-13+/+, SHIP+/+IL-13−/−, SHIP−/−IL-13+/+, and SHIP−/−IL-13−/− mice that were assessed at 8 weeks of age. An equal number of male and female mice from each genotype were included in all experiments. Mice were kept under specific pathogen-free conditions in sterilized filter-top cages with ad libitum access to autoclaved food and water at the Animal Care Facility at BC Children’s Hospital Research Institute (Vancouver, BC, Canada). Animal protocols were approved by the institutional Animal Care Committee and adhered to Canadian Council on Animal Care guidelines (Protocol #s A21-0035 and A24-0152).

### Macrophage culture and western blotting

Bone marrow macrophages were isolated from femora and tibiae of mice as previously described.^8^ Macrophages were cultured for 10 days, then stimulated with recombinant IL-4 or IL-13 (10 ng/mL; StemCell Technologies, Vancouver, BC, Canada) for 3 days to induce ArgI expression. For in vitro IL-4 blockade experiments, BMDMs were stimulated with IL-4 (10 ng/mL) with or without αIL-4 (1 μg/mL) for 15 minutes for pSTAT6 analysis or for 3 days for ArgI analysis. For arginase activity assay, SHIP+/+ and SHIP−/− BMDMs were unstimulated, or stimulated with recombinant IL-4 (10 ng/mL) or recombinant IL-13 (10 ng/mL) for 3 days. Whole-cell lysates for western blotting were prepared by lysing cells in 2 × Laemmli’s digestion mix, DNA was sheared with a 26-gauge needle, and lysates were boiled for 1 minute. Lysates were subjected to sodium dodecyl sulfate–polyacrylamide gel electrophoresis (SDS-PAGE) on a 10% polyacrylamide gel followed by western blotting. Antibodies used included anti-ArgI (D4E3M, cat# 93668, Cell Signaling Technologies), anti-p-STAT6 (phospho Y641) (EPR22599-52, cat# ab235591, Abcam) and anti-β-actin (13E5, cat# 4970S, Cell Signaling Technologies) as a loading control. All western blots were performed in 3 independent biological replicates. Normalized densitometry for repeated experiments is included in the corresponding figures.

### Intraperitoneal injection

At 6 weeks of age, SHIP−/− mice were injected intraperitoneally with either phosphate-buffered saline (PBS) (injection control), 25 mg/kg IgG isotype control (HRPN, cat# BE0088, BioXcell), or 25 mg/kg of an anti-IL-4 blocking antibody (BVD6-24G2, cat# BE0199, BioXCell) every other day until 8 weeks of age. For the IL-13 blockade, SHIP−/− mice received either PBS, 25 mg/kg of IgG isotype control (1CI35520, Genentech, San Francisco, United States), or 25 mg/kg of anti-IL-13 blocking antibody (1AH82571, Genentech, San Francisco, United States) until 8 weeks of age. After 14 days of treatment, mice were euthanized, and distal ilea and lungs were collected for analyses. Three mice per sex were used in each experimental group (*n* = 6).

### Histological analyses

Upon euthanasia, distal ilea were removed, cleared of contents, and fixed in PBS-buffered 10% formalin. In situ, lungs were perfused directly via the trachea to prevent airway collapse. A blunt-tip needle was inserted at the most superior position of the trachea to fill the lungs with PBS-buffered 10% formalin, as previously described.^8^ Lung tissue was then collected for histological analysis. Tissues were fixed at 4 °C for 24 hours and dehydrated in 70% ethanol before paraffin processing. For Alcian Blue–Periodic Acid-Schiff (AB-PAS) staining, tissues were dehydrated in 100% ethanol. Mouse and human tissue sections were embedded in paraffin and cut into 5-μm sections, which were stained with hematoxylin-eosin (H&E), Masson’s trichrome, or AB-PAS. Histological damage was scored using a 16-point scale by 2 individuals blinded to the experimental condition, as described previously.^8^ Assessment scoring included: loss of architecture, 0-4; immune cell infiltration, 0-4; goblet cell hyperplasia and hypertrophy, 0-2; ulceration, 0-2; edema, 0-2; and muscle thickening, 0-2. Collagen deposition was identified based on blue staining. For collagen quantification, Masson’s trichrome-stained sections were analyzed in ImageJ, and the collagen-positive area was measured as the proportion of total tissue area. Fibrosis scoring was assessed on Masson’s trichrome-stained ileal sections using a 6-point scale by a trained pathologist blinded to the experimental conditions, as described previously.^13^ Each sample was graded on a scale of 0 to 3 in 2 domains: collagen fiber disorganization/disarray and fibromuscular hyperplasia/muscularization/obliteration of the submucosa. Muscularis externa thickness was measured at 6 points in 10 cross sections separated by ≥ 50 μm. Immune cell infiltrates were counted in 6 representative fields of H&E-stained cross sections at 40 × magnification and 6 sections separated by ≥ 50 μm were counted for each mouse. The Alcian Blue/PAS–positive area was quantified using ImageJ and expressed as the proportion of total tissue area. Goblet cells per crypt-villus unit were counted in H&E-stained cross sections. For each mouse, goblet cell quantification was performed on 6 photographs per section from 6 uniform horizontal cross sections separated by ≥ 50 μm. The lung free space area was quantified using ImageJ and expressed as a proportion of the total lung area.

### Arginase activity assay

Tissues were homogenized in 1 mL lysis buffer (0.1% Triton X-100, 25 mM Tris pH 8, aprotinin [40 μg/mL], leupeptin [8 μg/mL], PMSF [100 μm]) using a Polytron MR2100 homogenizer. The homogenates were clarified by centrifuging at 13 000 × g for 10 minutes at 4 °C. Arginase activity was assessed by measuring the urea concentration resulting from the hydrolysis of L-arginine, as described previously.^8^

### Cytokine measurements

Cytokine concentrations in clarified full-thickness ileal tissue homogenates were quantified by ELISA. Kits for mouse IL-13 and IL-1β were from R&D Systems (Minneapolis, MN, United States), and kits for mouse IL-4 and TGF-β1 were from BD Biosciences (Mississauga, ON, Canada).

### Immunofluorescent staining

Ileal tissues were collected, fixed in PBS-buffered 10% formalin, embedded in paraffin, and sectioned at 5-μm thickness. Tissue sections were mounted on slides, dried, and prepared for staining. Slides were deparaffinized in xylene and rehydrated through graded ethanol to water. Antigen retrieval was performed in a steamer using citric acid–based antigen unmasking solution (Vector Laboratories, H-3300; pH 6.0) for 35 minutes. Sections were then permeabilized for 15 minutes in PBS containing 0.1% Triton X-100 and 0.05% Tween-20 and blocked for 1 hour at room temperature with 5% BSA in PBS containing 0.5% Triton X-100. Primary antibodies were diluted in blocking buffer supplemented with 5% normal serum matched to the host species of the secondary antibody. The following primary antibodies were used: anti-pSTAT6 (phosphoY641, Abcam, clone EPR22599-52, cat# ab235591), anti-vimentin (Thermo Fisher Scientific, clone 7X7H6, cat# MA5-35320), anti-Ki67 (Thermo Fisher Scientific, clone SolA15, cat# 14-5698-82), anti–c-Kit (R&D Systems, Human/Mouse CD117/c-Kit Antibody, cat# AF1356), and anti–mast cell tryptase (Abcam, clone AA1, cat# ab2378). Sections were incubated with primary antibodies overnight at 4 °C, washed in PBS containing 0.1% Triton X-100, and then incubated with species-appropriate fluorophore-conjugated secondary antibodies at room temperature in the dark. After additional washes in PBS containing 0.1% Triton X-100, slides were mounted using VECTASHIELD Vibrance Antifade Mounting Medium with DAPI (4′,6-diamidino-2-phenylindole; Vector Laboratories). Negative control sections stained without primary antibody were included to confirm staining specificity. Images were acquired using an Olympus BX61 microscope. For quantification, pSTAT6^ + ^cells, vimentin^+ ^cells, and vimentin^+ ^Ki67^+^ cells were counted in 3 randomly selected fields per section at 20 × magnification, whereas c-Kit^+ ^cells and tryptase^+ ^cells were counted in 6 randomly selected fields per section at 20 × magnification.

### Toluidine blue staining

Formalin-fixed, paraffin-embedded tissue sections (5 μm) were deparaffinized and rehydrated to distilled water. Sections were stained with 0.1% toluidine blue solution for 10 minutes, rinsed in distilled water, rapidly dehydrated through 95% and absolute alcohols, cleared in xylene, and mounted with synthetic resin. Mast cells were identified by metachromatic deep violet staining. For mouse tissues, toluidine blue–positive mast cells were quantified per tissue section and expressed as total mast cells per section. For human tissues, toluidine blue–positive mast cells were quantified and expressed as total mast cells per mm^2^ of tissue area.

### Single-cell RNA sequencing analyses

Single-cell RNA sequencing data were analyzed using Seurat. Data were normalized with SCTransform. Data were obtained from the study *Stricturing Crohn’s Disease Single-Cell RNA Sequencing Reveals Fibroblast Heterogeneity and Intercellular Interactions*^14^ and provided upon request.

### Human tissue samples

Formalin-fixed, paraffin-embedded human intestinal tissue samples were obtained from the CEGIIR biobank at the University of Alberta. Samples included healthy small intestine, diseased small intestine, and diseased colon tissues and were fixed in formalin for 24 hours prior to paraffin embedding and sectioning for downstream analyses.

### Bone marrow–derived mast cell generation and stimulation

Bone marrow cells were isolated from femora and tibiae of mice and cultured as previously described.^15^ Briefly, cells were maintained for 6 weeks in DMEM (Dulbecco’s Modified Eagle Medium) supplemented with 10% FBS, recombinant mouse IL-3 (10 ng/mL; Stemcell Technologies, Vancouver, BC, Canada; cat# 78042), 2 mM L-glutamine, 1% Antibiotic-Antimycotic (100X) (Thermo Fisher Scientific; cat# 15240062), and 50 μM β-mercaptoethanol to generate bone marrow–derived mast cells (BMMCs). Maturity of the BMMCs was confirmed by flow cytometry, and cells used for experiments were ≥ 99% c-Kit^ + ^FcεRIα^+^. For stimulation experiments, BMMCs were plated at a density of 1 × 10^6^ cells/mL. Cells were left unstimulated or stimulated with recombinant mouse IL-1β (10 ng/mL; Stemcell Technologies; cat# 78035), recombinant mouse IL-33 (100 ng/mL; PeproTech; cat# 210-33), or lipopolysaccharide (LPS) from *Escherichia coli* serotype 127: B8 (1 μg/mL; Sigma-Aldrich, St. Louis, MO, United States; cat# L3129) alone. For IgE sensitization conditions, cells were incubated overnight with anti-DNP IgE (2 μg/mL; Sigma-Aldrich; clone SPE-7, cat# D8406) alone or followed by stimulation with IL-1β, IL-33, or LPS. For IgE-mediated activation, cells were sensitized overnight with anti-DNP IgE and then stimulated with DNP-BSA (40 ng/mL; Invitrogen; cat# A23018) alone or in combination with IL-1β, IL-33, or LPS. Supernatants were collected and clarified by centrifugation at 500 × g for 5 minutes at 4 °C prior to downstream analyses.

### RNAscope in situ hybridization

RNAscope in situ hybridization was performed on formalin-fixed, paraffin-embedded mouse ileal sections and human intestinal sections, including healthy small intestine, diseased small intestine, and diseased colon, using the RNAscope Multiplex Fluorescent V2 Assay (Advanced Cell Diagnostics) according to the manufacturer’s instructions. Mouse tissues were processed using the standard protease-based workflow, including deparaffinization, hydrogen peroxide treatment, target retrieval, and digestion with RNAscope Protease Plus. *Il13* and *Mrgprb2* probes (Advanced Cell Diagnostics) were used in separate staining experiments. Human tissues were processed using a protease-free workflow and co-stained with an *Il13* probe (Advanced Cell Diagnostics) in combination with immunofluorescence staining for c-Kit detection, according to the manufacturer’s instructions. In all cases, signal was amplified according to the manufacturer’s protocol and detected with Opal 690. Sections were counterstained with DAPI and imaged using an Olympus BX61 microscope.

### Statistical analyses

Statistical analyses were conducted using GraphPad Prism version 10 (GraphPad Software, Inc, La Jolla, CA). Statistical differences between 2 groups were analyzed by an unpaired Student *t*-test and differences between multiple groups of data were analyzed by one-way analysis of variance (ANOVA) with Bonferroni’s correction. *P* < .05 was considered statistically significant.

## Results

### IL-13, but not IL-4, drives fibrosis in the distal ileum of SHIP−/− mice

We have previously shown that intestinal fibrosis in SHIP−/− mice depends on PI3Kp110δ activity and downstream ArgI^4^^,^^8^ which are activated downstream of IL-4 and IL-13 signaling. We therefore investigated whether IL-4 or IL-13 contributes to fibrosis in this model. To identify cytokines upstream of PI3Kp110δ-induced ArgI activity in SHIP−/− mice, we first examined whether IL-4 and IL-13 could induce ArgI in macrophages. In M-CSF-derived BMDMs, both IL-4 and IL-13 induced ArgI protein expression (Figure S1A) and arginase activity (Figure S1B), although IL-4 was the more potent stimulus. Moreover, SHIP−/− BMDMs were hyperresponsive to IL-4, inducing greater ArgI expression and activity compared to SHIP+/+ BMDMs, whereas IL-13 induced comparable ArgI protein expression and activity in SHIP+/+ and SHIP−/− BMDMs (Figure S1A, B). Additionally, IL-4 concentrations were increased in full-thickness ileal homogenates from SHIP−/− mice, whereas IL-13 concentrations were lower than in SHIP+/+ ilea (Figure S1C).

Based on these data, we initially hypothesized that IL-4 might contribute to fibrosis in SHIP−/− mice. To test this, SHIP+/+ and SHIP−/− mice were treated with PBS, IgG isotype control, or an IL-4 blocking antibody (αIL-4) from 6 to 8 weeks of age. Although αIL-4 effectively inhibited IL-4-induced pSTAT6 and ArgI expression in vitro in BMDMs (Figure S2A), it did not reduce pSTAT6-positive cells in the ilea of SHIP−/− mice in vivo (Figure S2B). Moreover, αIL-4 treatment failed to improve gross pathology (Figure S2C), histological damage (Figure S2D), collagen deposition (Figure S2E), fibrosis score, muscle thickening, arginase activity, or TGF-β1 concentrations in SHIP−/− ilea (Figure S2F). To exclude the possibility that incomplete neutralization accounted for this negative result, we next genetically ablated IL-4 in SHIP−/− mice. Similar to antibody blockade, IL-4 deficiency did not improve gross pathology (Figure S3A), histopathology (Figure S3B), collagen accumulation (Figure S3C), fibrosis score, muscle thickness, arginase activity, or TGF-β1 concentrations in SHIP−/− mice (Figure S3D).

**Figure 1:**
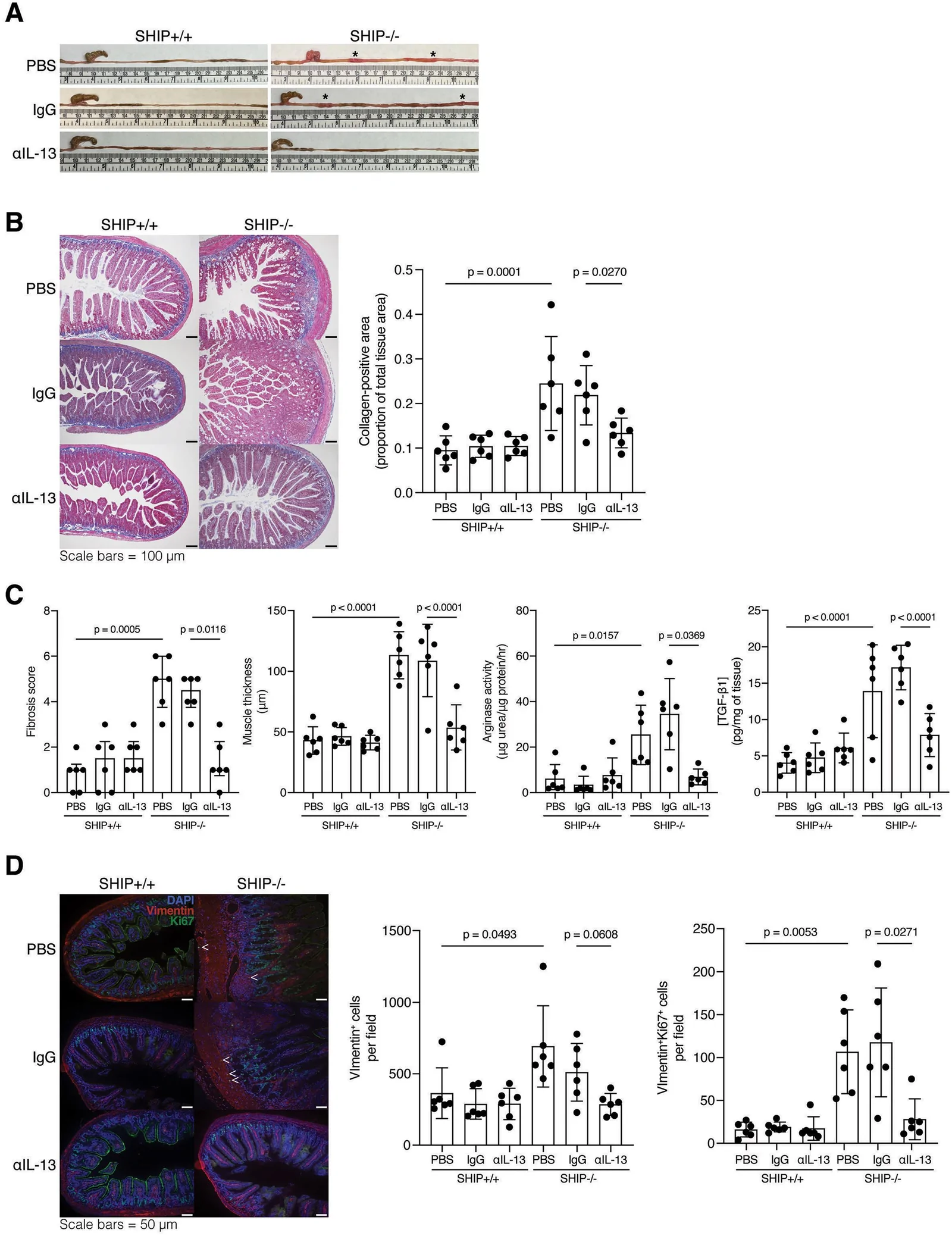
IL-13 blocking reduces fibrosis in SHIP−/− mice. SHIP+/+ and SHIP−/− mice were treated with either PBS, an IgG isotype control, or an IL-13 blocking antibody (αIL-13) by IP injection for 2 weeks from 6 to 8 weeks of age. (A) Gross anatomy of ceca and distal ilea were photographed. Asterisks indicate areas of thickening. Photographs are representative of *n* = 6 mice (3 male and 3 female) per group with similar results. (B) Fixed ileal cross sections were stained with Masson’s trichrome for fibrosis; collagen is stained blue. Photographs were taken at 10 × magnification; scale bars = 100 μm. Sections are representative of 6 mice (3 male and 3 female) per group with similar results. Collagen-positive area was quantified as the proportion of total tissue area. Data presented are means ± standard deviation (SD) for *n* = 6 mice (3 male and 3 female) per group. Statistical analysis was performed using one-way ANOVA followed by Bonferroni’s post-hoc test for multiple comparisons. (C) Fibrosis scores are expressed as median score with interquartile range (IQR) for *n* = 6 mice (3 male and 3 female) per group. Statistical analysis was performed using the Mann-Whitney test to compare ranks between groups with the Bonferroni adjustment for multiple comparisons. Muscle thickness was measured in H&E-stained ileal tissue cross sections from mice. For each mouse, muscle thickness was measured at 6 points on 10 uniform horizontal cross sections separated by ≥ 50 μm. Arginase activity and TGF-β1 concentrations were measured in full-thickness ileal tissue homogenates from mice. For muscle thickness, arginase activity and TGF-β1 concentrations, data presented are means ± SD for *n* = 6 mice (3 male and 3 female) per group and statistical analyses were conducted using a one-way ANOVA followed by Bonferroni’s post-hoc test for pairwise comparisons. (D) Ileal cross sections were stained by immunofluorescence using an anti-vimentin antibody (Thermo Fisher Scientific, clone 7X7H6, cat# MA5-35320) and an anti-Ki67 antibody (Thermo Fisher Scientific, clone SolA15, cat# 14–5698-82). Representative images are shown at 20 × magnification; scale bars = 50 μm. Arrows indicate Vimentin^ + ^Ki67^+^ (double-positive) cells. Vimentin^ + ^cells and double-positive cells were quantified in 3 randomly selected fields per section at 20 × magnification. Data are presented as mean ± SD from *n* = 6 mice per group (3 male and 3 female). Statistical analysis was performed using one-way ANOVA followed by Bonferroni’s post-hoc test for multiple comparisons. Differences were considered significant at *P* ≤ .05.

We therefore next investigated whether IL-13 contributes to ileal fibrosis in SHIP−/− mice. SHIP+/+ and SHIP−/− mice were treated with PBS, IgG isotype control, or an IL-13 blocking antibody (αIL-13) from 6 to 8 weeks of age. Whereas SHIP+/+ mice showed no evidence of pathosis regardless of treatment, SHIP−/− mice treated with PBS or IgG exhibited ileal thickening and red patches, both of which were reduced by αIL-13 treatment (Figure 1A). In addition, SHIP−/− mice displayed increased collagen deposition (Figure 1B), fibrosis scores, muscle thickening, arginase activity and TGF-β1 concentrations relative to SHIP+/+ controls (Figure 1C), and each of these fibrosis-associated features was reduced by αIL-13 treatment to amounts comparable to healthy SHIP+/+ mice (Figure 1B, C). To determine whether IL-13 blockade also affected expansion of fibroblasts, ileal cross sections from SHIP+/+ and SHIP−/− mice treated with PBS, IgG, or αIL-13 were stained for vimentin and Ki67. SHIP−/− mice exhibited increased numbers of total vimentin^ + ^cells and proliferating vimentin^ + ^Ki67^+^ cells compared to SHIP+/+ controls, and both populations were reduced in SHIP−/− mice by αIL-13 treatment (Figure 1D).

**Figure 2:**
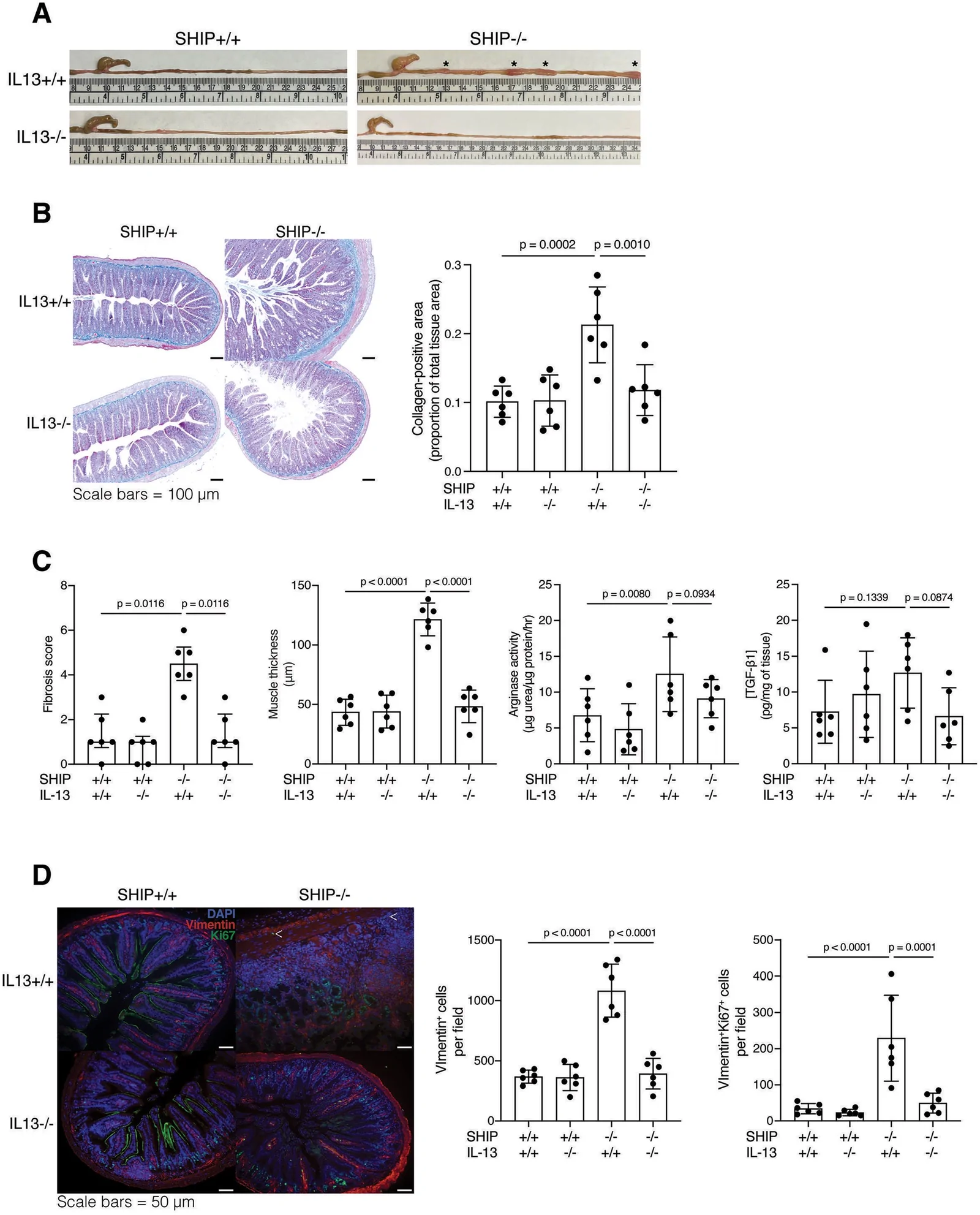
IL-13 deficiency reduces fibrosis in SHIP−/− mice. SHIP+/+IL-13+/+, SHIP+/+IL-13−/−, SHIP−/−IL-13+/+, and SHIP−/−IL-13−/− mice were euthanized at 8 weeks of age. (A) Gross anatomy of ceca and distal ilea were photographed. Asterisks indicate areas of thickening. Photographs are representative of *n* = 6 mice (3 male and 3 female) per genotype with similar results. (B) Fixed ileal cross-sections were stained with Masson’s trichrome for fibrosis; collagen is stained blue. Photographs were taken at 10 × magnification; scale bars = 100 μm. Sections are representative of 6 mice (3 male and 3 female) per genotype with similar results. The collagen-positive area was quantified as the proportion of total tissue area. Data presented are means ± SD for *n* = 6 mice (3 male and 3 female) per group. Statistical analysis was performed using one-way ANOVA followed by Bonferroni’s post-hoc test for multiple comparisons. (C) Fibrosis scores are expressed as median score with IQR range for *n* = 6 mice (3 male and 3 female) per group. Statistical analysis was performed using the Kruskal-Wallis test followed by Dunn’s multiple comparisons test. Muscle thickness was measured in H&E-stained ileal tissue cross sections from mice. For each mouse, muscle thickness was measured at 6 points on 10 uniform horizontal cross sections separated by ≥ 50 μm. Arginase activity and TGF-β1 concentrations were measured in full-thickness ileal tissue homogenates from mice. For muscle thickness, arginase activity and TGF-β1 concentrations, data presented are means ± SD for *n* = 6 mice (3 male and 3 female) per group and statistical analyses were conducted using a one-way ANOVA followed by Bonferroni’s post-hoc test for pairwise comparisons. (D) Ileal cross sections were stained by immunofluorescence using an anti-vimentin antibody (Thermo Fisher Scientific, clone 7X7H6, cat# MA5-35320) and an anti-Ki67 antibody (Thermo Fisher Scientific, clone SolA15, cat# 14–5698-82). Representative images are shown at 20 × magnification; scale bars = 50 μm. Arrows indicate Vimentin^ + ^Ki67^+^ (double positive) cells. Vimentin^ + ^cells and double-positive Vimentin^ + ^Ki67^+^ cells were quantified in 3 randomly selected fields per section at 20 × magnification. Data are presented as mean ± SD from *n* = 6 mice per group (3 male and 3 female). Statistical analysis was performed using one-way ANOVA followed by Bonferroni’s post-hoc test for multiple comparisons. Differences were considered significant at *P* ≤ .05.

To confirm a role for IL-13 in ileal fibrosis in SHIP−/− mice, SHIP−/− mice were crossed with IL-13 deficient mice (IL-13−/−) to produce SHIP+/+IL-13+/+, SHIP+/+IL-13−/−, SHIP−/−IL-13+/+, and SHIP−/−IL-13−/− mice. Mice were euthanized at 8 weeks of age and ileal pathology was examined. SHIP+/+IL-13+/+ and SHIP+/+IL-13−/− mice did not show any signs of gross pathology. SHIP−/−IL-13+/+ had enlarged ilea with patches of redness, and ileal pathology was eliminated by IL-13 deficiency (SHIP−/−IL-13−/− mice) (Figure 2A). Masson’s trichrome staining of ileal cross sections demonstrated increased collagen deposition in the distal ileum of SHIP−/−IL-13+/+ mice compared to SHIP+/+IL-13+/+ mice, which was significantly reduced by IL-13 deficiency in SHIP−/−IL-13−/− mice (Figure 2B). Fibrosis scores, muscle thickness, arginase activity, and TGF-β1 concentrations were elevated in SHIP−/−IL-13+/+ mice compared to SHIP+/+IL-13+/+ mice. Fibrosis scores and muscle thickness were reduced in SHIP−/−IL-13−/− mice (Figure 2C). Arginase activity and TGF-β1 concentrations were also lower in SHIP−/−IL-13−/− mice than in SHIP−/−IL-13+/+ mice, although these differences did not reach statistical significance (Figure 2C). To determine whether IL-13 deficiency affected fibroblast expansion, ileal cross sections from mice were stained for vimentin and Ki67. SHIP−/−IL-13+/+ mice had increased numbers of total vimentin^ + ^cells and proliferating vimentin^ + ^Ki67^+^ cells compared to controls, and both populations were reduced in SHIP−/−IL-13−/− mice (Figure 2D).

### Targeting IL-13 reduces ileal inflammation in SHIP-deficient mice

**Figure 3:**
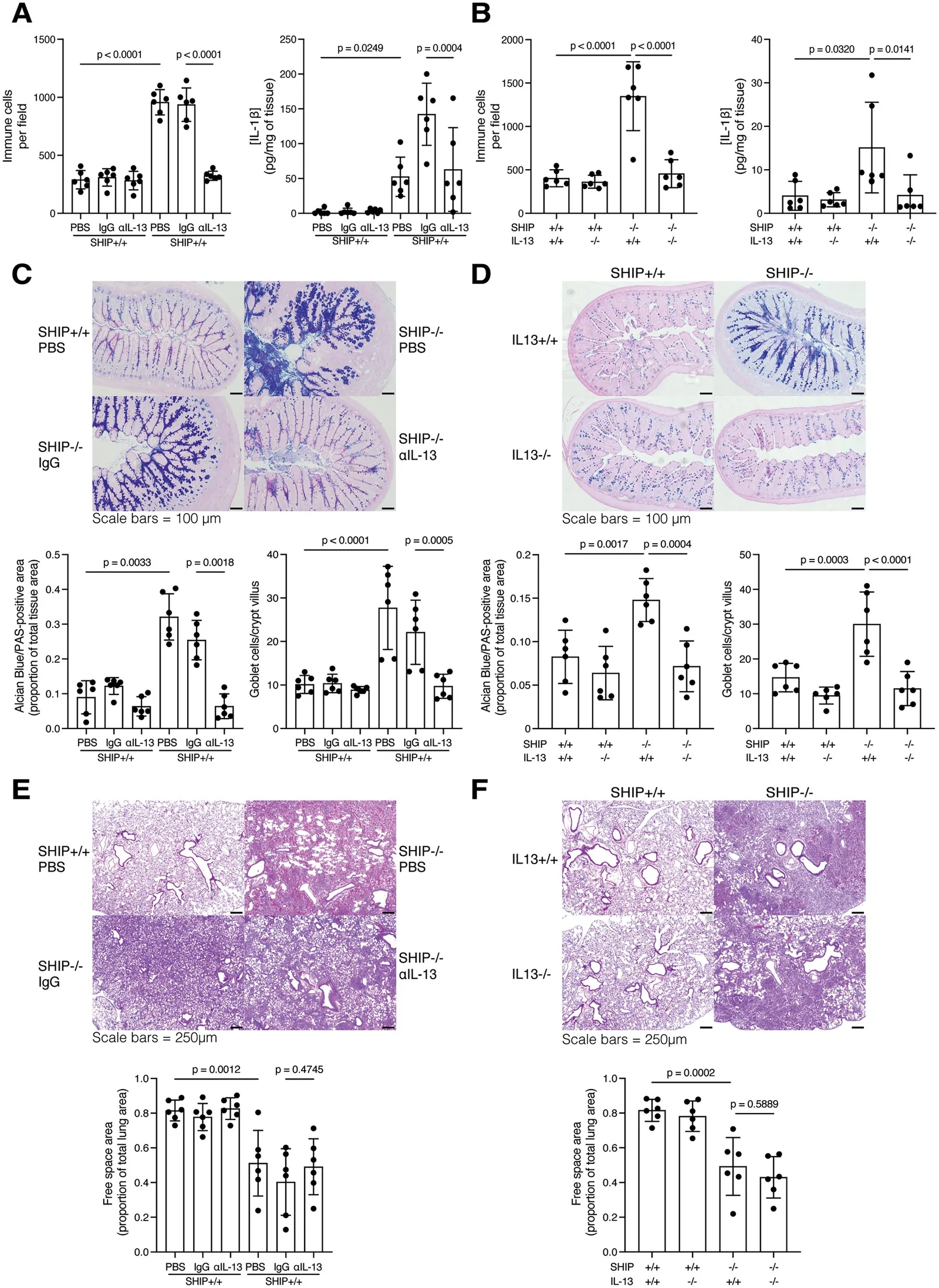
IL-13 blocking, and deficiency reduces inflammation in SHIP−/− mice. Inflammation was assessed in 8-week-old SHIP−/− mice by quantifying immune cell infiltrates in H&E-stained ileal cross sections and measuring IL-1β concentrations in full-thickness ileal tissue homogenates for SHIP−/− mice treated with αIL-13 (A) and SHIP−/−IL-13−/− mice (B). For each mouse, immune cell infiltrates were quantified from 6 photographs per section across 6 uniform horizontal cross sections separated by ≥ 50 μm. Data are presented as means ± SD for *n* = 6 mice (3 male and 3 female) per group. Statistical analysis was performed using one-way ANOVA followed by Bonferroni’s post hoc test for pairwise comparisons. Fixed ileal cross sections were stained with Alcian Blue/PAS for SHIP−/− mice treated with αIL-13 (C) and SHIP−/−IL-13−/− mice (D). Photographs were taken at 10 × magnification; scale bars = 100 μm. Sections are representative of *n* = 6 mice (3 male and 3 female) per group. Alcian Blue/PAS-positive area was quantified as the proportion of total tissue area. Goblet cells per crypt villus were counted in H&E-stained cross sections. For each mouse, quantitation was performed on 6 photographs per section for 6 uniform horizontal cross sections separated by ≥ 50 μm. Data presented are means ± SD for *n* = 6 mice (3 male and 3 female) per group. Statistical analyses were conducted using one-way ANOVA followed by Bonferroni’s post-hoc test for pairwise comparisons. Lungs were fixed and stained with H&E for SHIP−/− mice treated with αIL-13 (E) and SHIP−/−IL-13−/− mice (F). Representative images were acquired at 4 × magnification; scale bars = 250 μm. Sections shown are representative of 6 mice per group (3 male and 3 female) with comparable findings. Free space area was quantified as the proportion of total lung area. Data are presented as mean ± SD for n = 6 mice per group (3 male and 3 female). Statistical analysis was performed using one-way ANOVA followed by Bonferroni’s post hoc test for multiple comparisons. Differences were considered significant at *P* ≤ .05.

SHIP−/− mice treated with the IL-13 blocking antibody (Figure S4A) or SHIP−/− mice deficient in IL-13 (Figure S4B) had reduced histological damage scores, which include parameters that assess inflammation as well as fibrotic pathology. We therefore quantified inflammation in SHIP−/− mice treated with αIL-13 or deficient in IL-13. Immune cell numbers were higher in the ilea of SHIP−/− mice compared to SHIP+/+ mice. Immune cell infiltration was reduced by αIL-13 but not the IgG isotype control antibody (Figure 3A), and genetic ablation of IL-13 reduced immune cell numbers to amounts comparable to those seen in healthy SHIP+/+ mice (Figure 3B). We have previously reported that inflammation in SHIP-deficient mice is driven by macrophage-derived IL-1β^6^, and thus IL-1β was measured in full-thickness tissue homogenates from mice. Ileal IL-1β concentrations were significantly higher in SHIP−/− mice compared to SHIP+/+ mice (Figure 3A, B). Ileal IL-1β concentrations were reduced in SHIP−/− mice treated with αIL-13 compared to the IgG control (Figure 3A), and genetic ablation of IL-13 reduced ileal IL-1β concentrations in SHIP−/− mice to amounts measured in healthy SHIP+/+ mice (Figure 3B).

Given that goblet cell hyperplasia and hypertrophy are components of the histological damage score, which might be induced by IL-13 (a type 2 cytokine), we next assessed mucus-associated changes in the ileum. Ileal cross-sections from SHIP+/+ mice and SHIP−/− mice were stained with Alcian Blue/PAS, which stains mucins associated with type 2 inflammatory responses. The Alcian Blue/PAS-positive area was quantified, as well as goblet cells per crypt-villus unit. SHIP−/− mice had greater Alcian Blue/PAS-positive area, as well as more goblet cells per crypt-villus, compared to SHIP+/+ mice, and both parameters were reduced by treatment with αIL-13 (Figure 3C) and by IL-13 genetic deficiency (Figure 3D). Finally, lung pathology was examined because SHIP−/− mouse lungs display inflammatory pathology that is reduced by PI3Kp110δ deficiency.^8^ SHIP−/− mouse lungs were consolidated with immune cell infiltrates and had significantly less free space area compared to SHIP+/+ mice, but neither αIL-13 nor IL-13 deficiency reduced these features (Figure 3E, F).

### *IL13* is upregulated in stricturing Crohn disease and is localized to an expanded mast cell subcluster

**Figure 4:**
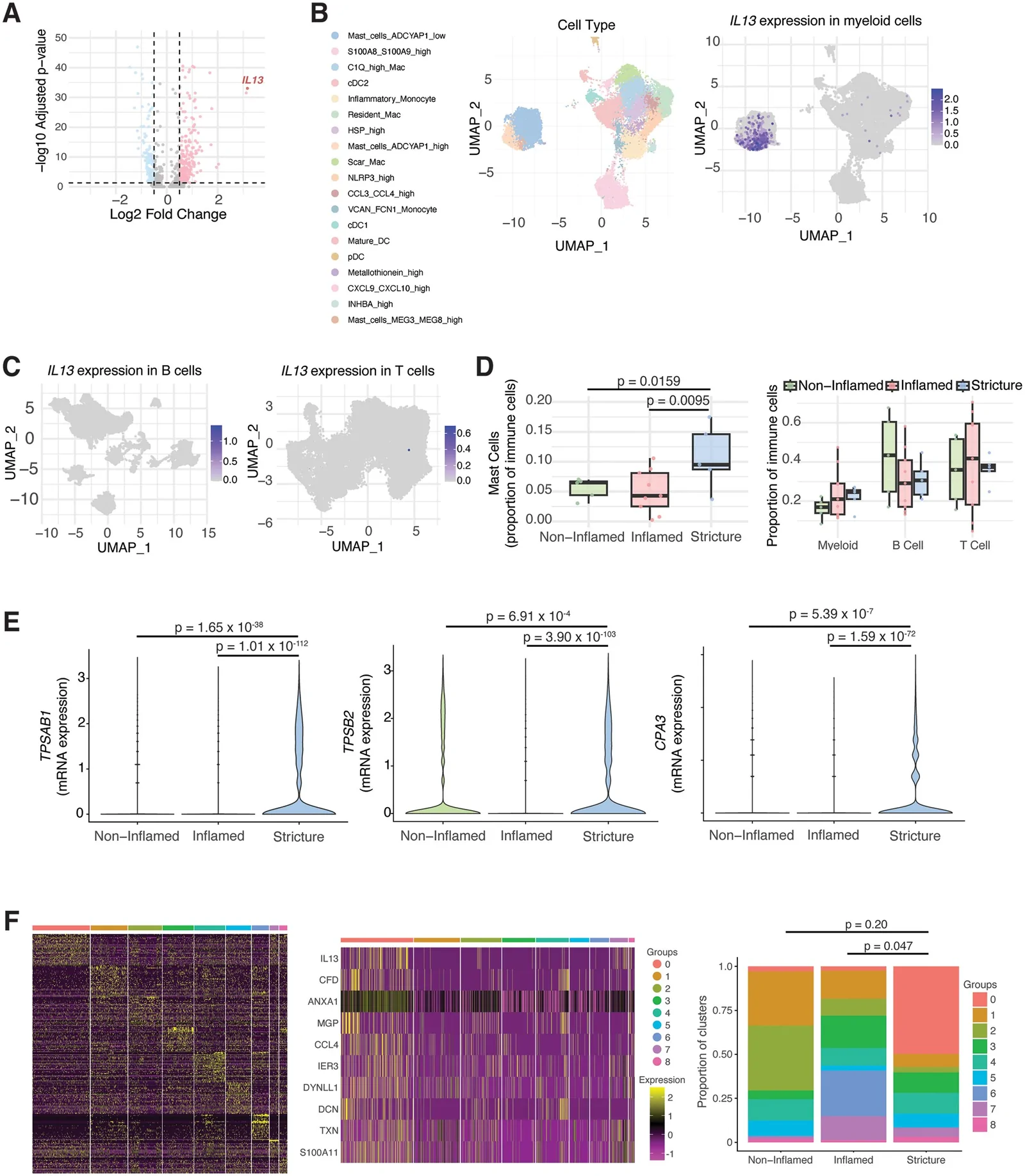
*IL13* is up-regulated in stricturing Crohn disease and mast cells are the source of *IL13*. *IL13* gene expression was examined in control, inflamed, and strictured ileal tissues from people with CD using single‐cell RNA sequencing data from the study “Stricturing Crohn’s Disease Single-Cell RNA Sequencing Reveals Fibroblast Heterogeneity and Intercellular Interactions” (data was provided upon request). (A) Differentially expressed genes between strictured (n = 5) and inflamed (n = 9) tissue sections in individuals with CD. Differential expression analysis was performed using Seurat’s FindMarkers function with a Wilcoxon rank-sum test, and *P* values were adjusted for multiple testing using the Benjamini-Hochberg correction. Each point represents a gene, with log_2_ fold change (Log_2_FC) on the x-axis and -log_10_(adjusted p-value) on the y-axis. Vertical dashed lines indicate the Log_2_FC threshold of ± 0.5, and horizontal dashed line represents the significance threshold of an adjusted *P* value of .05. Genes that do not meet significance criteria are shown in grey, significantly up-regulated genes in stricture tissue are shown in pink, and significantly down-regulated genes are shown in blue. *IL13* is highlighted in dark red. Data were normalized using SCTransform prior to analysis. (B) UMAP of myeloid cells, including mast cells, from non-inflamed (n = 5), inflamed (n = 9), and strictured (n = 5) tissue sections from individuals with CD. Each color representing a distinct myeloid cell subtype (left). *IL13* expression using a colour gradient, where higher expression is indicated by a more intense color (right). Data were processed using Seurat. Gene expression values were normalized using SCTransform. (C) UMAP visualization of B cells (left) and T cells (right) from non-inflamed (n = 5), inflamed (n = 9), and strictured (n = 5) tissue sections from individuals with CD gated on *IL13* expression. (D) Proportion of mast cells in non-inflamed (n = 5), inflamed (n = 9), and strictured (n = 5) tissue sections, calculated as a fraction of all immune cells per section (left). Statistical comparisons were performed using the Mann-Whitney U test (2-sided, unpaired). Proportion of myeloid cells (excluding mast cells), B cells, and T cells in the same tissue sections, shown as a proportion of all immune cells per section (right). (E) Violin plots displaying the expression levels of mast cell markers *TPSAB1*, *TPSB2*, and *CPA3* in non-inflamed, inflamed, and stricture ileal tissue sections. Data were plotted using the scaled data from the SCT assay. For each gene, expression was compared between stricture and inflamed, as well as stricture and non-inflamed groups, using the Wilcoxon rank-sum test. P-values were adjusted for multiple comparisons using the Bonferroni correction. (F) Mast cells subsets from the myeloid compartment were reclustered based on gene expression profiles using Seurat. Unsupervised clustering identified distinct mast cell subpopulations. A heatmap (left) shows the scaled expression of selected marker genes across mast cell clusters, highlighting a cluster characterized by high *IL13* expression (Cluster 0). The top 10 differentially expressed genes (ranked by log_2_ fold change) for Cluster 0 are shown in a heatmap to illustrate their expression across all mast cell clusters (middle). The proportion of each mast cell cluster within non-inflamed (n = 5), inflamed (n = 9), and strictured (n = 5) tissue sections was calculated as the fraction of total mast cells per section (right). Statistical comparisons between groups were performed using the Mann-Whitney U test (2-sided, unpaired). Differences were considered significant at *P* ≤ .05

To investigate the expression and source of *IL13* in people with CD, a single-cell RNA sequencing dataset derived from freshly collected full-thickness ileal sections from individuals with CD, encompassing uninflamed, inflamed nonstrictured, and strictured tissue segments were analyzed^14^ To determine whether *IL13* expression is upregulated in strictured tissue, differential gene expression analysis was performed to compare strictured tissue to inflamed tissue. Analysis revealed that *IL13* is the top upregulated gene in strictured tissue, with a significant fold change in expression relative to inflamed, nonstrictured tissue (Figure 4A). To determine the cellular source of *IL13* expression, the myeloid cell compartment was examined and *IL13* was exclusively expressed in mast cells, with no detectable expression in other myeloid cell types, including macrophages, dendritic cells, or neutrophils (Figure 4B). No detectable *IL13* expression was found in B cell and T cell datasets, including NK cells and ILCs (Figure 4C). Next, the relative abundance of mast cells in strictured tissue was assessed. The proportion of mast cells among the total immune cell population was quantified across non-inflamed, inflamed non-strictured, and strictured tissue sections. Mast cell proportions were significantly higher in strictured tissue compared to both non-inflamed and inflamed tissue sections (Figure 4D). In contrast, the proportions of other immune cell populations including myeloid cells (excluding mast cells), B cells, and T cells; did not show significant differences between the tissue sections (Figure 4D). To confirm increased numbers and proportions of mast cells, expression of mast cell-specific genes, including *TPSAB1*, *TPSB2*, and *CPA3*, was assessed. These genes were significantly upregulated in inflamed, strictured tissue compared to both non-inflamed and inflamed, non-strictured tissues (Figure 4E). To further characterize mast cell heterogeneity, mast cells were reclustered and analyzed separately, which identified nine distinct mast cell subclusters (groups) based on their gene expression profiles (Figure 4F). Examination of the top differentially expressed genes for subcluster 0 revealed that *IL13* was the most upregulated gene in this cluster. A heat map of the top 10 upregulated genes in subcluster 0 demonstrated that this transcriptional program was enriched specifically within subcluster 0 relative to the other mast cell subclusters (Figure 4F). To determine whether particular mast cell subsets were associated with tissue state, the proportional abundance of each mast cell subcluster was compared across non-inflamed, inflamed nonstrictured, and strictured tissue sections. This analysis showed that subcluster 0, the *IL13*-expressing mast cell population, was significantly enriched in strictured tissue compared to both noninflamed and inflamed nonstrictured tissue (Figure 4F).

### Fibrotic small intestinal tissue from people with IBD contains increased mast cells that colocalize with *IL13*

**Figure 5:**
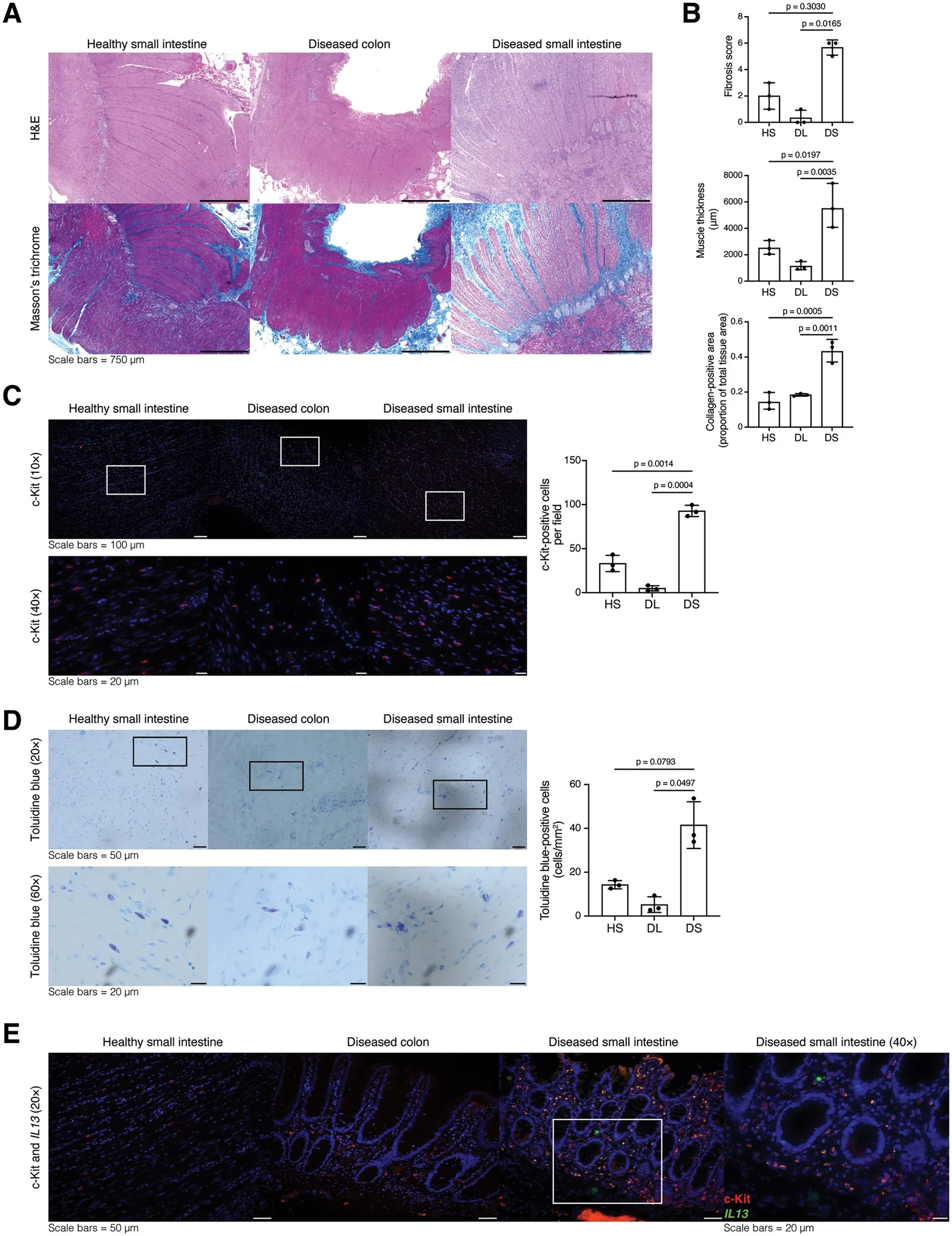
Mast cells accumulate in strictured intestinal tissue from individuals with IBD and represent a source of *IL13*. Healthy small intestine, diseased colon, and diseased small intestine samples were obtained from surgical resections (*n* = 3 per group). (A) Serial cross sections of surgical resection samples were stained with H&E (top row) and Masson’s trichrome (bottom row) to assess tissue architecture and fibrosis. Photographs were taken at 4 × magnification; scale bars = 750 μm. Sections are representative of 3 independent samples per group with similar results. (B) Fibrosis scores are expressed as median score with interquartile range for *n* = 3 per group. Statistical analysis for fibrosis scores were performed using the Kruskal-Wallis test followed by Dunn’s multiple comparisons test. Muscle thickness was measured in H&E-stained ileal tissue cross sections. For each section, muscle thickness was measured at 10 points. Collagen deposition was quantified from Masson’s trichrome-stained sections by measuring the collagen-positive area as a proportion of total tissue area. Muscle thickness and collagen deposition are presented as mean ± SD. Statistical analyses for muscle thickness and collagen deposition were conducted using one-way ANOVA followed by Bonferroni’s post-hoc test for pairwise comparisons. (C) Ileal cross sections were stained by immunofluorescence using an anti-c-Kit antibody (Human/Mouse CD117/c-Kit Antibody, R&D Systems, cat# AF1356). Representative images are shown at 10 × magnification (top; scale bars = 100 μm) and 40 × magnification (bottom; scale bars = 20 μm). C-Kit^ + ^cells were quantified in 6 randomly selected fields per section at 20 × magnification and are presented as c-Kit^ + ^cells per field. Data are presented as mean ± SD. Statistical analysis was performed using the Kruskal-Wallis test, followed by Dunn’s multiple comparisons test. (D) Mast cells were identified by toluidine blue staining of cytoplasmic granules. Representative images are shown at 20 × magnification (top; scale bars = 50 μm) and 60 × magnification (bottom; scale bars = 20 μm). Toluidine blue-positive cells were quantified and expressed as total mast cells per mm^2^ of tissue area. Data are presented as mean ± SD. Statistical analysis was performed using the Kruskal-Wallis test, followed by Dunn’s multiple comparisons test. (E) Tissue sections were stained for *IL13* by RNAscope, followed by c-Kit immunostaining, to identify the cellular source of *IL13*. Representative images are shown at 20 × magnification (scale bars = 50 μm) and 40 × magnification (far right panel; scale bar = 20 μm). Red arrow heads denote c-Kit^ + ^cells and orange arrow heads denote c-Kit^ + ^*IL13*^+^ cells. Images are representative of 3 independent samples per group with similar results. Differences were considered significant at *P* ≤ .05.

To determine whether mast cells are associated with fibrosis in human IBD tissue, resection specimens from healthy small intestine, diseased colon, and diseased small intestine were examined. H&E- and Masson’s trichrome–stained cross sections showed marked architectural distortion and abundant collagen accumulation in diseased small intestine compared to healthy small intestine and diseased colon (Figure 5A). Fibrosis was then quantified by histological fibrosis score, muscle thickness, and collagen-positive area. Fibrosis scores were significantly higher in diseased small intestine compared to diseased colon, although the difference between healthy small intestine and diseased small intestine did not reach statistical significance (Figure 5B). In contrast, both muscle thickness and collagen-positive area were significantly increased in diseased small intestine compared to both healthy small intestine and diseased colon (Figure 5B).

To assess mast cell abundance, tissue sections were stained by immunofluorescence for c-Kit. Diseased small intestine contained significantly more c-Kit^ + ^cells than both diseased colon and healthy small intestine, with c-Kit^ + ^staining predominantly localized to the intestinal muscle layers and with additional cells present in the submucosa (Figure 5C). Mast cells were also assessed using toluidine blue staining, which stains cytoplasmic granules. Consistent with the c-Kit data, toluidine blue–positive cells were significantly increased in diseased small intestine compared to diseased colon and were also higher in diseased small intestine compared to healthy small intestine, although the latter difference did not reach statistical significance (Figure 5D). Similar to c-Kit staining, toluidine blue–positive cells were predominantly localized to the muscle layers.

To investigate whether mast cells are a source of *IL13* in human intestinal tissue, cross-sections were analyzed by RNAscope for *IL13* transcripts followed by immunofluorescence staining for c-Kit to identify mast cells. Mast cells were identified in all tissue types; however, *IL13* signal was detectable only in diseased small intestine. In these tissues, a subset of c-Kit^ + ^cells colocalized with *IL13*, thereby identifying mast cells as a source of *IL13* in strictured small intestinal resections (Figure 5E).

### Mast cells accumulate in the distal ileum of SHIP−/− mice and contribute to *Il13* production

**Figure 6:**
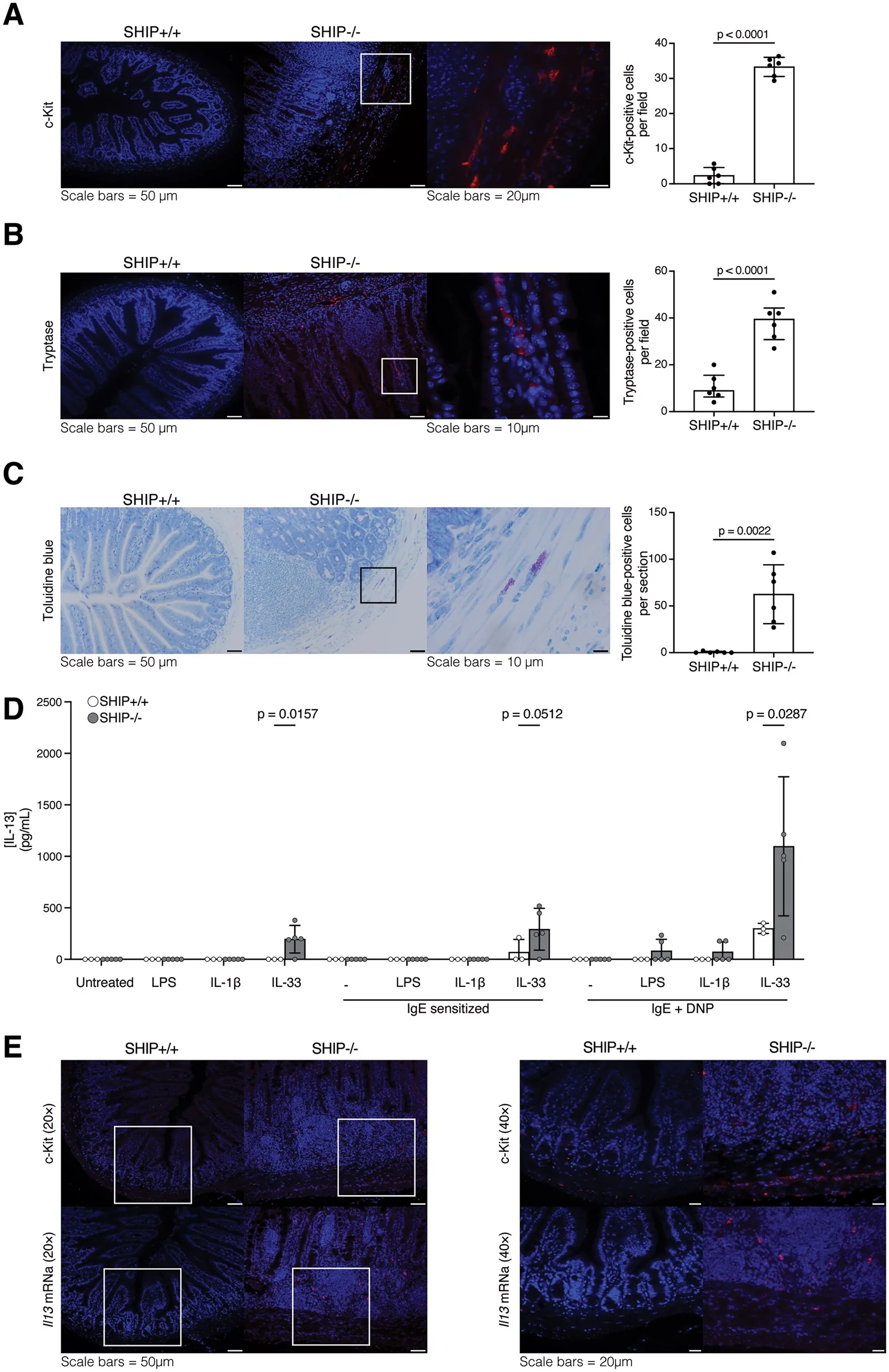
Mast cell numbers are increased in SHIP−/− mice and mast cells represent a source of *Il13*. (A) Ileal cross sections from SHIP+/+ and SHIP−/− mice were stained by immunofluorescence using an anti-c-Kit antibody (Human/Mouse CD117/c-Kit Antibody, R&D Systems, cat# AF1356) to identify mast cells. Representative images are shown at 20 × magnification (left and middle; scale bars = 50 μm) and 60 × magnification (right; scale bar = 20 μm). C-Kit^ + ^cells were quantified in 6 randomly selected fields per section at 20 × magnification and are presented as c-Kit^ + ^cells per field. Data are presented as mean ± SD for *n* = 6 mice per group (3 male and 3 female). Statistical analysis was performed using one-way ANOVA followed by Bonferroni’s post-hoc test for multiple comparisons. (B) Ileal cross sections from SHIP+/+ and SHIP−/− mice were stained by immunofluorescence using an anti-mast cell tryptase antibody (Abcam, clone AA1, cat# ab2378). Representative images are shown at 20 × magnification (left and middle; scale bars = 50 μm) and 100 × magnification (right; scale bars = 10 μm). Tryptase^ + ^cells were quantified in 6 randomly selected fields per section at 20 × magnification and are presented as tryptase^ + ^cells per field. Data are presented as mean ± SD for *n* = 6 mice per group (3 male and 3 female). Statistical analysis was performed using one-way ANOVA followed by Bonferroni’s post-hoc test for multiple comparisons. (C) Mast cells were identified by toluidine blue staining of cytoplasmic granules in ileal cross sections from SHIP+/+ and SHIP−/− mice. Representative images are shown at 20 × magnification (left and middle; scale bars = 50 μm) and 100 × magnification (right; scale bars = 10 μm). Toluidine blue-positive mast cells were quantified per tissue section and are presented as total mast cells per section. Data are presented as mean ± SD for *n* = 6 mice per group (3 male and 3 female). Statistical analysis was performed using one-way ANOVA followed by Bonferroni’s post-hoc test for multiple comparisons. (D) Bone marrow–derived mast cells (BMMCs) were generated by culturing bone marrow cells for 6 weeks in the presence of IL-3. BMMCs were left unstimulated or stimulated with LPS, IL-1β, or IL-33 under 3 conditions: without sensitization, following overnight IgE sensitization, or following IgE sensitization and DNP stimulation. Data are presented as mean ± SD for BMMCs derived from *n* = 3 SHIP+/+ and *n* = 5 SHIP−/− mice. Statistical analysis was performed using Welch’s t test for each comparison. (E) Serial ileal tissue sections from SHIP+/+ and SHIP−/− mice were stained by immunofluorescence for c-kit (top row) and by RNAscope for *Il13* (bottom row) to assess the spatial relationship between mast cells and *Il13* mRNA expression. Representative images are shown at 20 × magnification (left; scale bars = 50 μm) and 40 × magnification (right; scale bars = 20 μm). Differences were considered significant at *P* ≤ .05.

To determine whether mast cells are increased in the distal ileum of SHIP−/− mice, ileal cross sections from SHIP+/+ and SHIP−/− mice were stained for c-Kit. SHIP−/− mice had significantly more c-Kit^ + ^cells than SHIP+/+ mice (Figure 6A). Similar to human tissue, the majority of c-Kit^ + ^staining in SHIP−/− ilea was localized to the muscle layers, with additional positive cells observed at the tips of the villi (Figure 6A). To confirm these findings using an additional mast cell marker, ileal cross sections were stained for tryptase. Consistent with the c-Kit staining, SHIP−/− mice had significantly more tryptase^+ ^cells than SHIP+/+ mice, with staining again localized primarily to the muscle layers and villus tips (Figure 6B). Mast cells were also assessed by toluidine blue staining, which likewise showed significantly increased mast cell numbers in SHIP−/− ilea compared with SHIP+/+ ilea. As toluidine blue staining depends on the presence of mast cell granules, it may be less sensitive for detecting degranulated mast cells, while partially degranulated mast cells may stain less intensely, thereby underrepresenting true mast cell numbers. Toluidine blue^+ ^cells were confined to the muscle layers and were not observed at the villus tips (Figure 6C).

To determine whether mast cells from SHIP−/− mice produce IL-13, SHIP+/+ and SHIP−/− bone marrow–derived mast cells (BMMCs) were left unstimulated or stimulated with LPS, IL-1β, or IL-33, either alone, following IgE sensitization, or following IgE sensitization and 2,4-dinitrophenylhydrazine (DNP) cross-linking. IL-13 production was measured by ELISA. IL-33 stimulated IL-13 production by SHIP−/− BMMCs under all 3 conditions: without sensitization, following IgE sensitization alone, and following IgE sensitization plus DNP stimulation, and was dramatically upregulated higher when cells were costimulated with IL-33 and IgE cross-linking (Figure 6D). In contrast, SHIP+/+ mast cells only produced detectable levels of IL-33 when costimulated with IL-33 and IgE or IL-33 and (IgE + DNP). Moreover, SHIP−/− BMMCs produced substantially more IL-13 than SHIP+/+ mast cells in response to IL-33, indicating that SHIP−/− mast cells are hyperresponsive to IL-33 (Figure 6D).

To determine whether mast cells are a source of *Il13* in vivo in SHIP−/− mice, serial ileal sections from mice were stained by immunofluorescence for c-Kit or by RNAscope to detect *Il13* transcripts. *Il13* mRNA was present within the muscle layer coincident with mast cells, but also observed within immune cell infiltrates in the submucosa, suggesting that mast cells are not the only source of *Il13* in SHIP−/− mice (Figure 6E). To further define the mast cell phenotype in SHIP−/− mice, serial ileal cross sections were stained by immunofluorescence for c-Kit or by RNAscope to detect *Mrgprb2*, a marker of connective tissue mast cells.^16^ Across the distal ileum of SHIP−/− mice, *Mrgprb2* signals were observed only rarely, and these were consistently confined to the muscle layer (Figure S5A). Given the abundance of mast cells identified throughout the muscle layer and the relative paucity of *Mrgprb2* staining, these findings indicate that the vast majority of mast cells in the SHIP−/− distal ileum exhibit a mucosal mast cell phenotype (Figure S5A).

## Discussion

Using antibody-mediated blockade and genetic ablation, we demonstrate that IL-13, but not IL-4, is a critical driver of intestinal fibrosis in SHIP-deficient mice. Targeting IL-13 also reduced intestinal inflammation, indicating that in this model IL-13 contributes not only to fibrotic remodeling but also to inflammatory disease. Extending these findings to human disease, analysis of single-cell RNA sequencing data from people with CD showed that *IL13* is upregulated in strictured tissue and is expressed exclusively by mast cells within the immune compartment.^14^ We further identified an *IL13*-high mast cell subcluster enriched specifically in strictured tissue and validated these findings in human resections, where mast cells were increased in diseased small intestine and identified as a predominant source of *IL13*. In parallel, mast cells were also increased in SHIP−/− ilea and contributed to *Il13* production.

Although IL-4 and IL-13 share receptor components and overlapping downstream signaling pathways, they can exert distinct biological effects.^17^ This distinction is particularly important in the SHIP−/− model, where IL-4 is a stronger inducer of ArgI protein and enzyme activity in macrophages, yet blockade or loss of IL-13, and not IL-4, reduced fibrosis.^4^ These findings suggest that IL-13 promotes intestinal fibrosis through mechanisms that extend beyond macrophage ArgI induction and likely involve effects on nonhematopoietic target cells. This interpretation is consistent with established roles for IL-13 in epithelial and stromal remodeling, including goblet cell hyperplasia, mucus production, and tissue fibrosis in other organs.^17^^,^^18^ Notably, these features are prominent in SHIP−/− mice and are reduced following IL-13 blockade, supporting the idea that IL-13 acts as an effector cytokine driving pathological remodeling.

Our findings also help clarify why IL-13 appears pathogenic in SHIP−/− mice despite the limited success of IL-13–targeted therapies in UC. Clinical trials of IL-13 blockade in UC have not shown meaningful benefit.^19^^,^^20^ This finding may reflect differences in disease biology, as UC is not primarily a fibrostenotic disease and is characterized by a heterogeneous inflammatory milieu involving multiple immune pathways, including IL-23/IL-17–associated responses, rather than a simple type 2-dominant program.^21^^,^^22^ In contrast, SHIP−/− mice develop chronic ileal inflammation together with marked fibrotic remodeling and characterized by a type 2 inflammatory response, in which IL-13 plays a pathogenic role. In this context, IL-13 may contribute directly to both tissue remodeling and barrier dysfunction, such that its inhibition reduces fibrosis as well as secondary inflammation. Consistent with this context dependence, IL-13 deficiency does not protect against fibrosis in chronic dextran sulfate sodium (DSS)–induced colitis, an epithelial injury–driven model in which tissue damage is initiated independently of a type 2 immune response.^23^ By contrast, other intestinal fibrosis models provide indirect or partial support for a role for IL-13. SAMP1/YitFc mice develop chronic ileitis and fibrosis. In SAMP1/YitFc mice, early ileitis is driven by Th1 cytokines, whereas chronic ileitis is characterized by a shift toward Th2 cytokines, accompanied by fibrosis and elevated *Il13* expression.^24^ Although a direct requirement for IL-13 has not been tested, anti–IL-4 treatment reduces ileal pathology in this model.^24^ In addition, losartan treatment reduces ileitis and fibrosis in SAMP1/YitFc mice that is associated with reduced *Il13* expression.^25^ Similar correlative evidence exists in TL1A-overexpressing mice, in which fibrotic pathology is accompanied by increased IL-13 production.^26^ The most direct complementary preclinical evidence comes from chronic TNBS (2,4,6-trinitrobenzenesulfonic acid) colitis, in which interfering with IL-13 signaling reduces collagen accumulation by limiting IGF-1-dependent myofibroblast activation.^27^ Together, these comparisons suggest that IL-13 is not a universal driver of intestinal fibrosis but instead has context-dependent pathogenic effects that are most relevant in settings of fibrostenotic remodeling and type 2 immune activation.

An unexpected finding in this study was that inhibition or deletion of IL-13 also reduced intestinal inflammation in SHIP−/− mice. Because type 1 and type 2 immune pathways cross-regulate one another, one might predict that reducing IL-13 would exacerbate inflammation.^9^ Instead, our data suggest that in this model the net effect of IL-13 is proinflammatory. One possible explanation is that IL-13 contributes to epithelial barrier dysfunction. Unlike IL-4, IL-13 has been shown to reduce transepithelial resistance in intestinal epithelial monolayers^28^ raising the possibility that an IL-13 blockade improves barrier integrity and thereby limits downstream inflammation. More broadly, the anti-inflammatory effect of an IL-13 blockade is consistent with our previous finding that inhibition of PI3Kp110δ reduces both fibrosis and inflammation in SHIP−/− mice,^8^ supporting a model in which IL-13 signals through the type 2 receptor to engage downstream pathways that promote both tissue remodeling and inflammatory pathology. These findings may also help explain why fibrosis can persist despite control of overt inflammation in CD. While inflammation may initiate fibrotic responses, established fibrosis may in turn perpetuate local inflammatory signaling.

Our human data identify mast cells as a major cellular source of *IL13* in fibrostenotic CD. Although mast cell expansions in CD strictures were reported >25 years ago, their functional contribution has remained unclear.^29^ Here, we show that mast cell expansion in strictured tissue is accompanied by a distinct transcriptional program, rather than simply reflecting increased cell numbers. In particular, reclustering identified an *IL13*–high mast cell subpopulation selectively enriched in strictured tissue. Histologic analysis further showed that mast cells are increased in diseased small intestine, localized predominantly to the muscle layers where fibrotic remodeling is most evident, and are a major source of *IL13.* These findings strongly support the idea that mast cells are not merely bystanders in chronic inflammation but may actively contribute to fibrostenotic pathology through local IL-13 production. This idea is consistent with prior literature reporting that IL-13 can be produced by several immune cell types, including mast cells.^30^

The SHIP−/− model recapitulated several key aspects of this mast cell signature. Mast cells were significantly increased in the distal ileum of SHIP−/− mice, and analyses of serial sections showed that a subset of *Il13* transcripts localized to the muscle layer, supporting the conclusion that mast cells contribute to *Il13* production in vivo in SHIP-deficient mice, although they are not the only source of *Il13*. This interpretation is strengthened by our in vitro findings that SHIP−/− bone marrow–derived mast cells produce robust amounts of IL-13 in response to IL-33 and do so to a much greater extent than SHIP+/+ mast cells, indicating that SHIP-deficient mast cells are hyperresponsive to IL-33. Given that SHIP is a well-established negative regulator of mast cell activation, these findings suggest that increased mast cell abundance together with heightened responsiveness may amplify IL-13 production in the diseased SHIP−/− ileum.^31^

We were unable to directly test whether mast-cell–derived IL-13 is required for fibrosis in SHIP−/− mice because systemic mast cell depletion using anti-CD117 caused rapid lethality. This result likely reflects the well-described role of SHIP as a gatekeeper of mast cell degranulation.^31^ SHIP-deficient mast cells exhibit a lower threshold for activation, and systemic c-Kit targeting likely provokes catastrophic degranulation and anaphylaxis.^31^ As a result, future studies will require mast cell–directed genetic approaches that avoid c-Kit–dependent systemic effects. Conditional deletion of IL-13 in mast cells or inducible mast cell depletion systems could provide a more precise framework to determine whether mast cells, and specifically mast-cell–derived IL-13, are required for intestinal fibrotic remodeling in SHIP−/− mice.^32^^,^^33^

These findings have potential therapeutic implications. Fibrosis in CD has long been viewed as a largely irreversible consequence of chronic inflammation, yet our data identify several potentially targetable nodes in this pathway. Upstream, IL-13 itself may represent a tractable therapeutic target, particularly in fibrostenotic CD or in molecularly defined subgroups characterized by low SHIP activity. More broadly, our results suggest that targeting signaling pathways downstream of the IL-13/type 2 receptor may also have anti-fibrotic potential. In this regard, JAK inhibitors are of particular interest because the type 2 receptor signals through JAK family kinases, and emerging clinical and translational data suggest that these agents may modulate fibroblast-associated programs in CD.^34^^,^^35^ It will be important to determine whether patients receiving these therapies show reduced development or progression of fibrostenotic complications.

Finally, our findings may have relevance for patient stratification. We, and others, have shown that a subset of people with CD have low SHIP activity,^6^^,^^36^ and reduced SHIP activity has been associated with more severe disease and greater need for surgery related to fibrotic complications.^37^ The current study extends these observations by identifying IL-13 as a functionally important downstream effector in SHIP-deficient intestinal fibrosis and by implicating mast cells as a key source of this cytokine in both human strictures and SHIP−/− mice. Together, these data support a model in which mast-cell–derived IL-13 promotes fibrostenotic remodeling in a context-dependent manner and suggest that this pathway may be particularly relevant to a subgroup of people with CD predisposed to fibrotic complications.

## Supplementary Material

izag127_Supplementary_Data

## Data Availability

Data, methods, and study materials will be made available to researchers upon request.
