# Supplementary material for: Mast cell–derived IL-13 as a driver of Crohn disease–associated intestinal fibrosis
Source: Inflamm Bowel Dis. 2026 Jul 8;32(9):1783–99. doi: 10.1093/ibd/izag127 (PMC13533194; doi:10.1093/ibd/izag127)
Supplement: Supplementary file 1 [file izag127_supplementary_data.pdf]

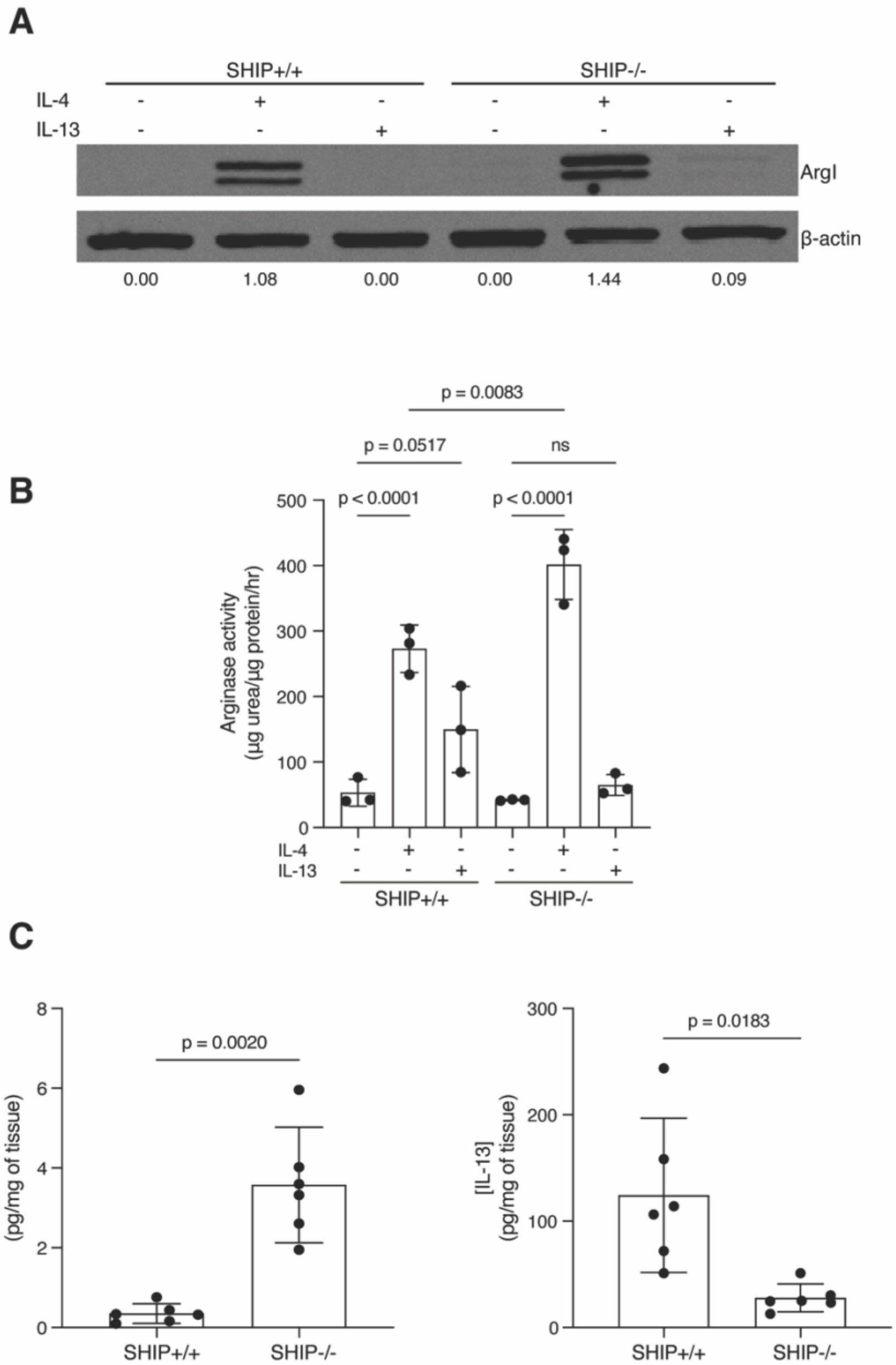

Supplementary Figure 1. Safari et al., 2026

Supplementary Figure 1. IL-4 and IL-13 induce ArgI expression and arginase activity in bone marrow derived macrophages. (A) SHIP+/+ and SHIP-/- BMDMs were unstimulated or stimulated with IL-4 or IL-13 for 3 days. ArgI protein was assessed by western blotting using an anti-ArgI antibody (Cell Signaling Technologies, D4E3M, cat# 93668) and β-actin (Cell Signalling Technologies, 13E5, cat# 4970), as a loading control. Data are representative of three biological replicates with similar results. Average densitometry values are listed. (B) Arginase activity. Data presented are means ± SD for bone marrow derived macrophages (BMDMs) from n = 3 mice per genotype assessed in three independent experiments. Data were analyzed using a two-way ANOVA. Post-hoc pairwise comparisons were performed using Bonferroni's correction for multiple comparisons. (C) IL-4 and IL-13 concentrations in full-thickness ileal tissue homogenates from 8-week-old mice. Data are means ± SD for n = 6 mice (three male and three female). Data were analyzed using a Welch's t test. Differences were considered significant at $p \leq .05$; ns = not significantly different.

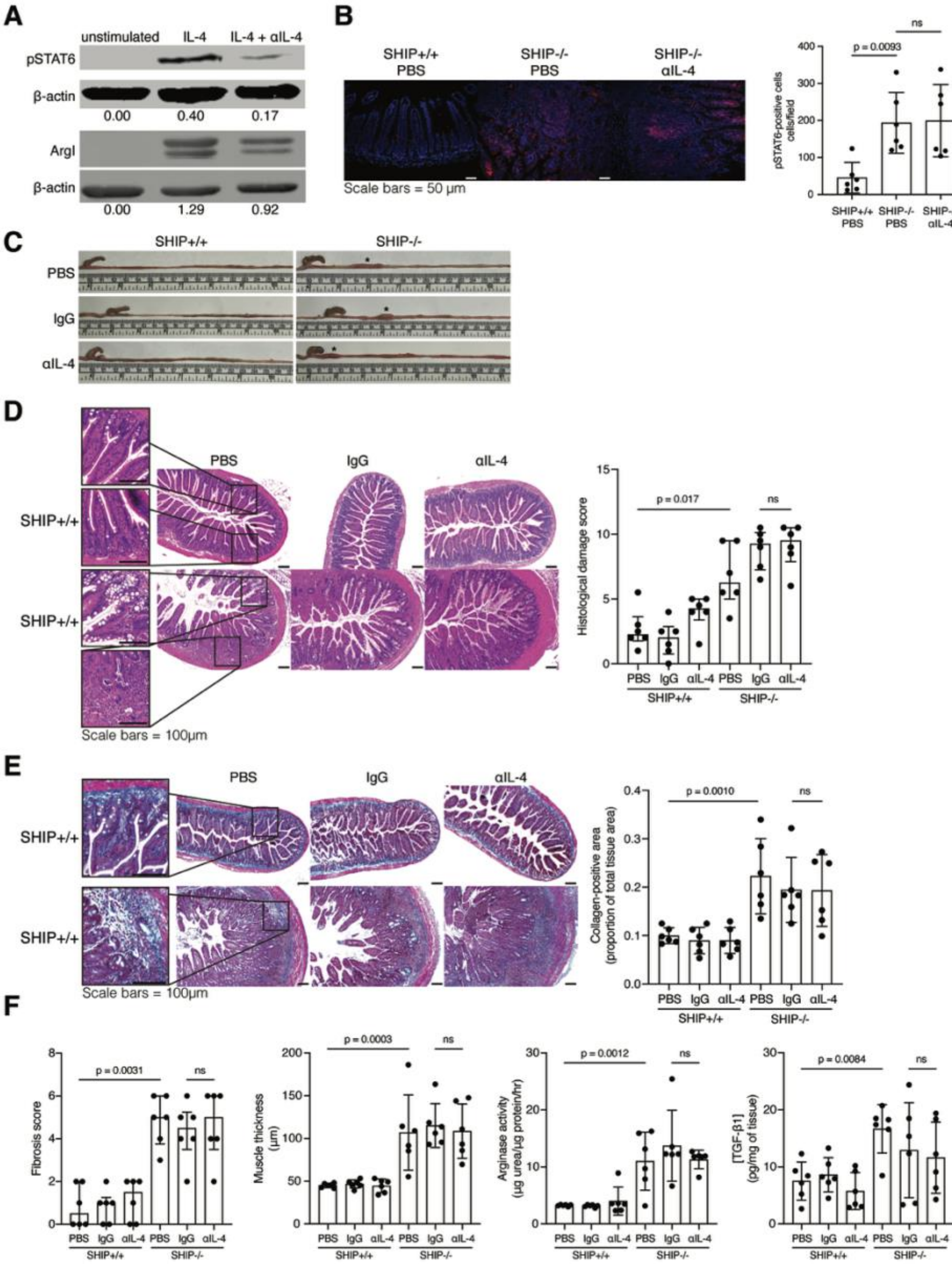

Supplementary Figure 2. Safari et al., 2026

Supplementary Figure 2. IL-4 blocking did not reduce fibrosis in SHIP-/- mice. (A) SHIP+/+ BMDMs were unstimulated or stimulated with IL-4 in the presence or absence of the IL-4 blocking antibody (αIL-4). Phospho-STAT6 (pSTAT6) and ArgI protein were assessed by western blotting using an anti-pSTAT6 (phosphoY641) and anti-ArgI antibody (Abcam, EPR22599-52, cat# ab235591 and Cell Signaling Technologies, D4E3M, cat# 93668) and β-actin (Cell Signalling Technologies, 13E5, cat# 4970), as a loading control. Data shown is representative of three biological replicates with similar results. Average densitometry values are listed. (B) Ileal cross sections from SHIP+/+ and SHIP-/- mice treated with PBS or αIL-4 were stained by immunofluorescence using an anti-pSTAT6 (phosphoY641) antibody (Abcam, clone EPR22599-52, cat# ab235591). Representative images are shown at 20× magnification; scale bars = 50 µm. pSTAT6-positive cells were quantified in three randomly selected fields per section at 20× magnification. Statistical analysis was performed using one-way ANOVA followed by Bonferroni's post-hoc test for multiple comparisons. Data presented are means ± SD for n = 6 mice (three male and three female) per group. (C) SHIP+/+ and SHIP-/- mice were treated with either PBS, an IgG isotype control, or αIL-4 by IP injection for 2 weeks from 6 to 8 weeks of age. Gross anatomy of ceca and distal ilea were photographed. Photographs are representative of n = 6 mice (three male and three female) per group. Asterisks indicate areas of thickening. (D) H&E-stained ileal cross sections from mice were photographed at 10× magnification; scale bars = 100 µm. Insets for each image show crypt-villus architecture and goblet cells (top) and immune cells/immune cell infiltration (bottom). Sections are representative of n = 6 mice (three male and three female) per group. Histological damage score for SHIP+/+ and SHIP-/- mice are expressed as median score with interquartile range for n = 6 mice (three male and three female) per group. Statistical analysis was performed using the Mann-Whitney

test to compare ranks between groups with Bonferroni adjustment for multiple comparisons. (E) Fixed ileal cross sections were stained with Masson's trichrome for fibrosis; collagen is stained blue. Photographs were taken at 10× magnification; scale bars = 100 µm. Insets show collagen deposition (blue) in the submucosa; scale bars = 100 µm. Sections are representative of six mice (three male and three female) per group. Collagen-positive area was quantified as the proportion of total tissue area. Data presented are means ± SD for n = 6 mice (three male and three female) per group. Statistical analysis was performed using one-way ANOVA followed by Bonferroni's post-hoc test for multiple comparisons. (F) Fibrosis scores for SHIP+/+ and SHIP-/- mice are expressed as median score with interquartile range for n = 6 mice (three male and three female) per group. Statistical analysis was performed using the Mann-Whitney test to compare ranks between groups with the Bonferroni adjustment for multiple comparisons. Muscle thickness was measured in H&E-stained ileal tissue cross sections from mice. For each mouse, muscle thickness was measured at 6 points on 10 uniform horizontal cross sections separated by ≥50 µm. Arginase activity and TGF-β1 concentrations were measured in full-thickness ileal tissue homogenates from mice. For muscle thickness, arginase activity and TGF-β1 concentrations, data presented are means ± SD for n = 6 mice (three male and three female) per group and statistical analyses were conducted using a one-way ANOVA followed by Bonferroni's post-hoc test for pairwise comparisons. Differences were considered significant at $p \leq .05$; ns = not significantly different.

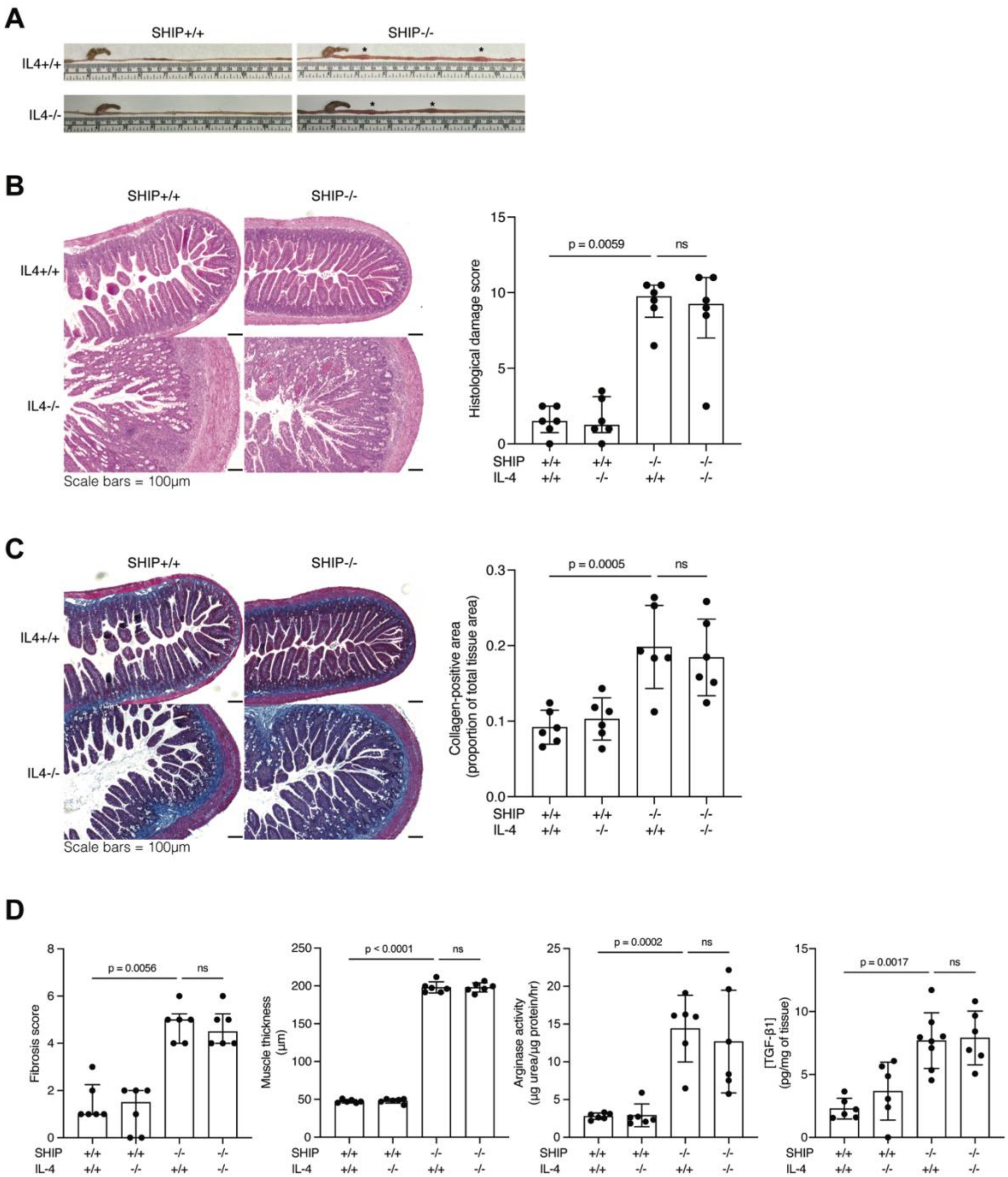

Supplementary Figure 3. Safari *et al.*, 2026

Supplementary Figure 3. IL-4 deficiency did not reduce fibrosis in SHIP-/- mice.

SHIP+/+IL-4+/+, SHIP+/+IL-4-/-, SHIP-/-IL-4+/+, and SHIP-/-IL-4-/- mice were euthanized at 8-weeks of age. (A) Gross anatomy of ceca and distal ilea were photographed. Asterisks indicate areas of thickening. Data shown are representative of 6 mice (three male and three female) per group with similar results. (B) H&E-stained ileal cross sections from mice were photographed at 10× magnification; scale bars = 100 µm. Histological damage is expressed as median score ± interquartile range. Statistical analysis was performed using the Kruskal-Wallis test, followed by Dunn's multiple comparisons test. (C) Fixed ileal cross sections were stained with Masson's trichrome for fibrosis; collagen is stained blue. Photographs were taken at 10× magnification; scale bars = 100 µm. Insets show collagen deposition (blue) in the submucosa; scale bars = 100 µm. Sections are representative of six mice (three male and three female) per genotype. Collagen-positive area was quantified as the proportion of total tissue area. Data presented are means ± SD for n = 6 mice (three male and three female) per group. Statistical analysis was performed using one-way ANOVA followed by Bonferroni's post-hoc test for multiple comparisons. (D) Fibrosis scores are expressed as median score with interquartile range for n = 6 mice (three male and three female) per group. Statistical analysis was performed using the Kruskal-Wallis test followed by Dunn's multiple comparisons test. Muscle thickness was measured in H&E-stained ileal tissue cross sections from mice. For each mouse, muscle thickness was measured at 6 points on 10 uniform horizontal cross sections separated by ≥50 µm. Arginase activity and TGF-β1 concentrations were measured in full-thickness ileal tissue homogenates from mice. For muscle thickness, arginase activity and TGF-β1 concentrations, data presented are means ± SD for n = 6 mice (three male and three female) per group and statistical analyses were conducted using a one-way ANOVA followed by Bonferroni's post-hoc

test for pairwise comparisons. Differences were considered significant at $p \leq .05$; ns = not significantly different.

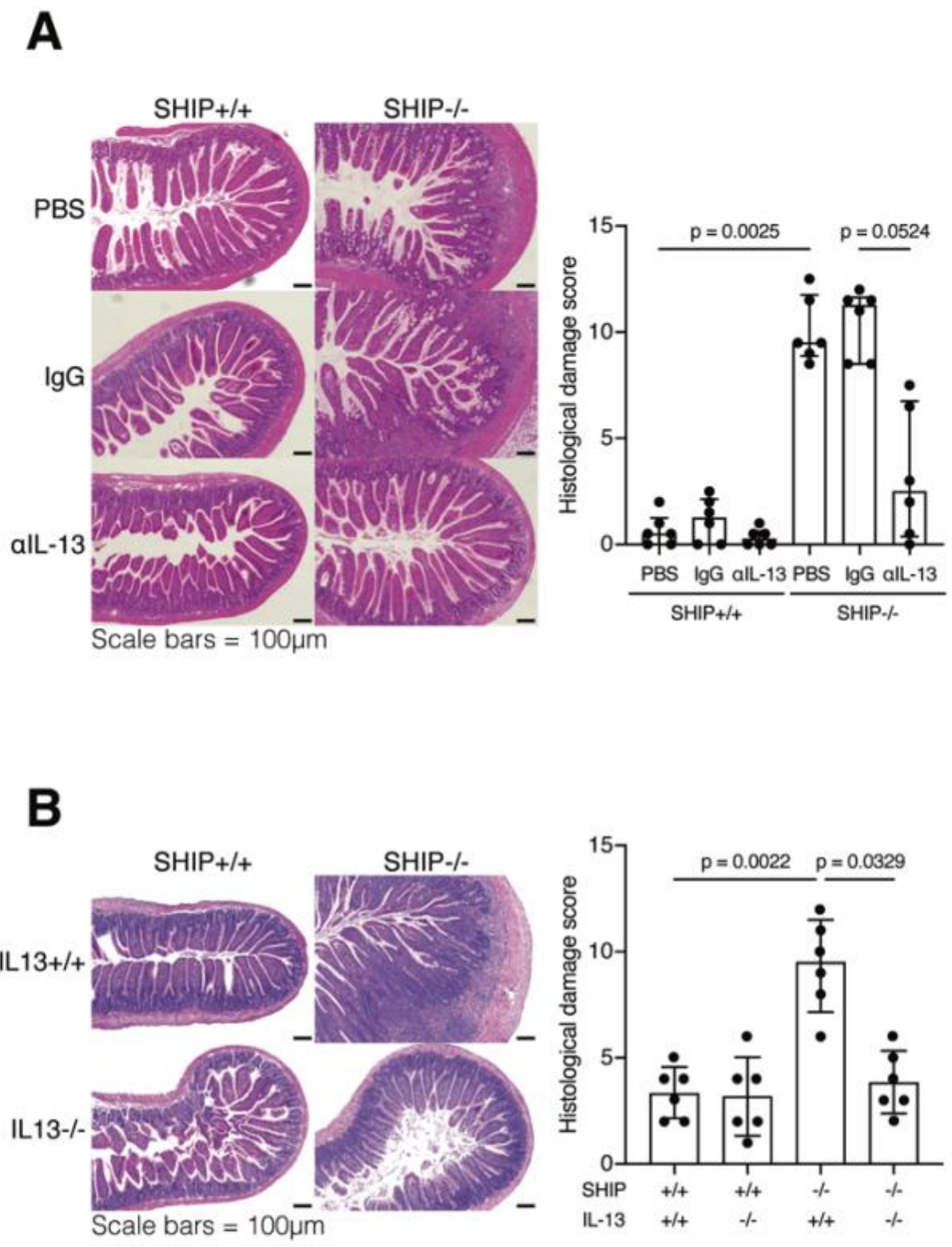

Supplementary Figure 4. Safari *et al.,* 2026

Supplementary Figure 4. IL-13 blockade and genetic deficiency reduce histopathological damage in SHIP-/- mice. H&E-stained ileal cross sections were photographed at 10× magnification; scale bars = 100 µm. (A) Histopathological damage in SHIP-/- mice treated with IL-13 blockade. (B) Histopathological damage in SHIP-/-IL-13-/- mice. Histological damage scores are presented as median with interquartile range. Sections are representative of n = 6 mice (three male and three female) per group with similar results. Statistical analysis for (A) was performed using the Mann-Whitney test with Bonferroni adjustment for multiple comparisons, and for (B) using the Kruskal-Wallis test followed by Dunn’s multiple comparisons test.

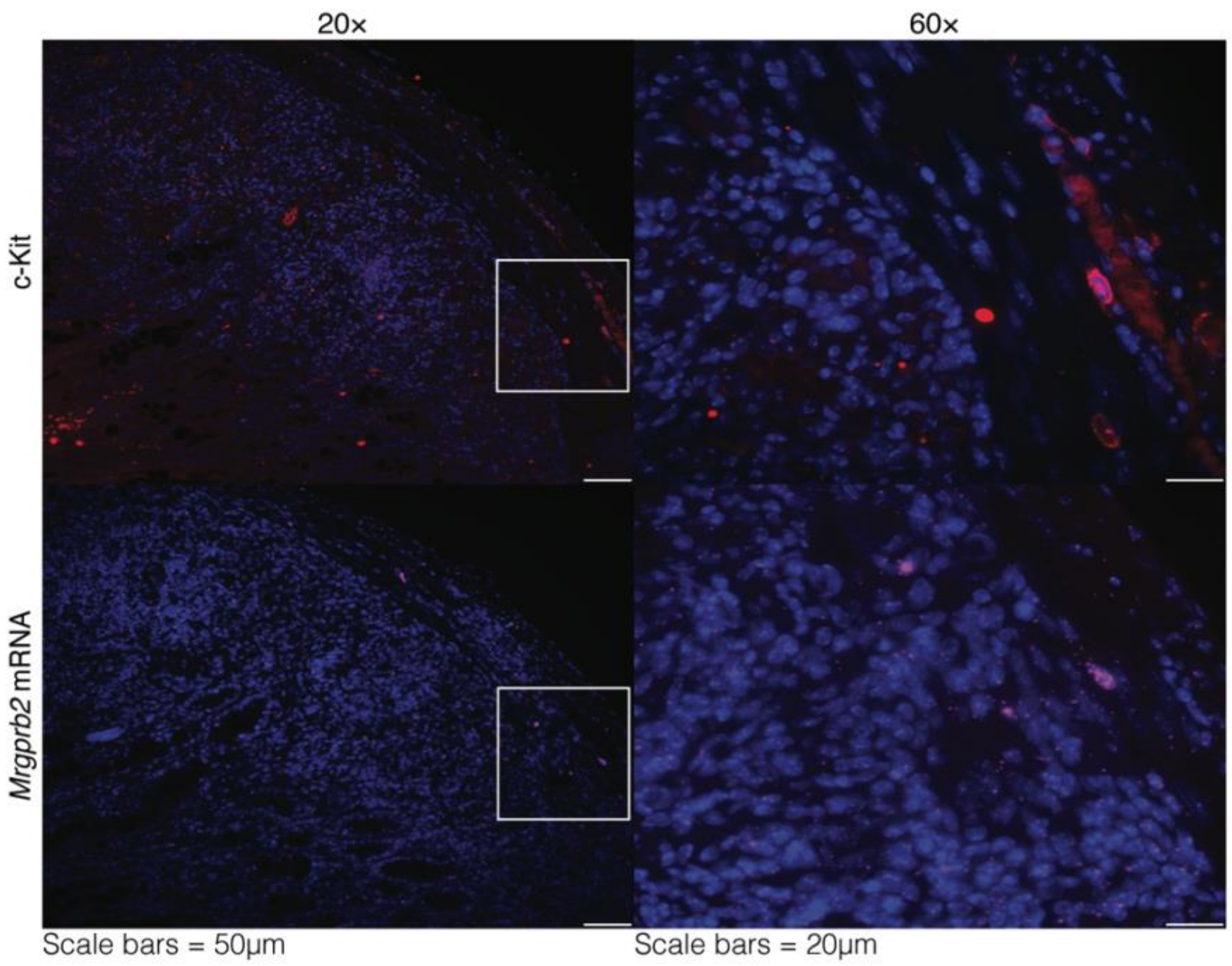

Supplementary Figure 5 Safari *et al.*, 2026

Supplementary Figure 5. The majority of mast cells in the SHIP-/- distal ileum exhibit a mucosal mast cell phenotype. Serial ileal tissue sections from SHIP-/- mice were stained by immunofluorescence for c-Kit (top row) and by RNAscope for *Mrgprb2* (bottom row) to determine whether mast cells exhibited a mucosal mast cell phenotype (*Mrgprb2*-negative) or a connective tissue mast cell phenotype (*Mrgprb2*-positive). Representative images are shown at 20× magnification (left; scale bars = 50 µm) and 60× magnification (right; scale bars = 20 µm).
